# The cost-effectiveness of a 20% price discount on fruit, vegetables, diet drinks and water, trialled in remote Australia to improve Indigenous health

**DOI:** 10.1371/journal.pone.0204005

**Published:** 2018-09-27

**Authors:** Anne Magnus, Linda Cobiac, Julie Brimblecombe, Mark Chatfield, Anthony Gunther, Megan Ferguson, Marj Moodie

**Affiliations:** 1 Deakin Health Economics, Centre for Population Health Research, Faculty of Health, Deakin University, Geelong, Victoria, Australia; 2 Burden of Disease Epidemiology, Equity and Cost-Effectiveness Programme, Department of Public Health, University of Otago, Wellington, New Zealand; 3 Wellbeing and Preventable Chronic Diseases Division, Menzies School of Health Research, Royal Darwin Hospital Campus, Tiwi, Northern Territory, Australia; 4 Wellbeing and Preventable Chronic Diseases Division, Menzies School of Health Research, Brisbane, Queensland, Australia; 5 Global Obesity Centre, Centre for Population Health Research, Faculty of Health, Deakin University, Geelong, Victoria, Australia; University of Waterloo, CANADA

## Abstract

This paper estimates the cost-effectiveness of a 20% price discount on healthy food and beverages with and without consumer nutrition education, as trialled in remote Northern Australia. Changes in actual store sales, from the pre-discount baseline period, were analysed for population impact on consumption of fruit and vegetables, water and artificially sweetened soft drinks, in addition with total dietary weight (grams), energy (Mega Joules), and sodium (milligrams). Disability Adjusted Life Years (DALYs), arising from changes in dietary risk factor prevalence in the population, were estimated as the primary health outcome in a multi health-state Markov model. The costs of the strategies were sourced from paid invoices and time estimates of staff providing store-based discount promotion and consumer education. The incremental cost-effectiveness ratio adopted a partial societal perspective, (including health and retail sector costs), as cost per DALY averted and was presented in 2011 Australian dollars. The price discount, helped address a gap in food price equity for residents of remote communities. However, the discount strategy, with or without consumer education led to a net loss of population health –36 95%CI (-47,-25) or -21(-28, -15) DALYs respectively, at increased cost to the retail and health sectors, of AUD860000 95%CI (710000, 1million) or AUD500000 (410000, 590000). The strategies trialled were thereby categorised as dominated by current practice while acknowledging considerable uncertainty surrounding the health outcome estimates. The 20% discount on limited targeted products appeared to need to be considered in conjunction with other marketing strategies to support healthy food choices, if remote Australian Indigenous population health is to be improved.

## Introduction

Poor diet is a leading behavioural cause of ill health as reported in the Global Burden of Disease estimates [1]. Low intake of fruit and vegetables is a leading modifiable contributor to that avoidable burden. Disadvantaged groups facing higher economic stress, tend to have poorer health and diets [2]. Aboriginal and Torres Strait Islander (hereafter, Indigenous) Australians living in remote communities experience both socio-economic and geographic disadvantage, with limited access to competitive shopping choices, and facing higher food prices (up to 60% higher) [3].

A growing body of research is investigating the promotion of healthier diets through the provision of financial incentives or subsidies to encourage higher consumption of healthier foods [4–7]. The economic viability of such strategies and their impact on population health are important information to provide decision makers considering their introduction. Previous modelling work which involved a number of important assumptions indicated the promising nature of fiscal strategies in this population [8]. The major assumption requiring testing in a real life situation was the impact on total dietary weight following discounting. Studies that have included other populations experiencing disadvantage have shown similar promising results [4–6, 9]. No trial-based cost effectiveness studies of fiscal strategies to improve diet have been conducted in Australian Indigenous populations [8, 10].

A trial, Stores Healthy Options Project in Remote Indigenous Communities (SHOP@RIC), used a stepped-wedge design, in 20 remote Indigenous communities where captured store sales data represented 96% (range 55%-119%) of the community populations’ daily energy requirements. The protocol, baseline diet and primary outcomes of the SHOP@RIC study have been published [11, 12]. A secondary aim of SHOP@RIC was to assess the cost-effectiveness of the fiscal strategies thus determining if the discount strategies represented good value-for-money in relation to health outcomes measured as Disability Adjusted Life Years (DALYs) averted over the lifetime of the trial-based population.

The health outcome modelling incorporates the significant evidence of selected avoidable risk factors on mortality and morbidity. Excess Body Mass Index (BMI) [13, 14] is a risk factor for ischaemic heart disease (IHD), ischaemic stroke, diabetes [15] and colon and breast cancers [16]. Increased servings of fruit and vegetables separately are associated with protective effects against IHD [17], stroke [18] and colon cancer [19]. Changes in sodium intake are associated with heart disease and stroke through changes in blood pressure [20, 21].

It was hypothesised that during the 20% price discount period (24 weeks), 1) an increase in consumption of fruit and vegetables would occur and healthier beverages would displace unhealthy beverages, 2) health gains (modelled as DALYs averted) over the remaining lifetime of the trial population would be positive and 3) this trial-based model could demonstrate that the fiscal strategies would offer value-for-money in this context. This paper presents the economic evaluation of the 20% discount strategy, with and without an in-store consumer education program, as implemented in the SHOP@RIC trial.

## Methods

### Setting

Cost-effectiveness analysis was conducted alongside the SHOP@RIC trial [11] in accordance with economic evaluation reporting guidelines [22]. Data on the effectiveness and cost of two fiscal strategies were combined to determine whether they represented potential value-for-money for future adoption in the remote Indigenous Australian setting, where community food stores are largely owned by community representatives.

### Target population

The target population comprised 8,515, (children 41% adults 59%) living in 20 remote Indigenous communities of Northern Territory in Australia [23], where the store was the principal supply of food and beverages consumed in the community. The communities met criteria for remoteness from other shopping outlets [11].

### Interventions

Two fiscal strategies were offered over a period of 24 weeks: (i) 20% discount on fresh fruit, fresh and frozen vegetables, artificially sweetened soft drinks and bottled water in 10 communities; (ii) 20% discount on fresh fruit, fresh and frozen vegetables, artificially sweetened soft drinks and bottled water combined with an in-store consumer education program in a second 10 communities. Communities were randomly allocated to a strategy and a starting date, in groups of four, with eight week intervals (steps) between starting dates, to enable each community to receive the intervention supported by relevant promotional and educational materials at each store location (i.e. a stepped wedge controlled trial design) [11].

The trial was registered on the Australian New Zealand Clinical Trials Registry ACTRN12613000694718. Ethics approval was provided by the combined Northern Territory Department of Health and Menzies School of Health Research Human Research Ethics Committee (HREC-2012-1711), the Central Australian Human Research Ethics Committee (HREC-2012-13 HREC-2012-1711) and Deakin University Human Research Ethics Committee (HREC-2012-243 HREC-2012-1711). Written informed consent was received from each of the participating community store boards after presentation and discussion of the project with each board.

### Control comparator

The two strategies were compared at 24 weeks (end of the discount period) and 48 weeks follow-up (i.e. 24 weeks after the end of the discount period) to usual practice with seasonal and secular trends accounted for. Usual practice comprised weekly sales data collected during the 49-week period, prior to the trial (i.e. no-discounts or consumer education strategy) [11].

### Perspective

The analysis adopted a partial societal sector perspective combining that of the health sector and commercial stores (that offered and promoted the price discounts and provided the venue where the consumer education took place).

### Outcomes

Intermediate dietary changes per person per day, arising from actual purchases of 26 categories of food and beverage lines [8], were assessed as total grams of dietary weight (gm), mega joules (MJ) of total energy, and milligrams (mg) of sodium intake. Own and cross-price elasticities in response to the price change of fruit, vegetables, diet soft drinks and water were calculated as the percent change in baseline quantity demanded (grams) over the 24-week discount period. Final health outcomes were measured in DALYs averted as a result of increases in grams of fruit and vegetable consumption, dietary sodium and estimated impact on average population BMI over 12 months.

#### Intermediate outcomes-diet

Percentage changes (mean and standard error) during the discount period and the follow-up period, by intervention, were calculated by the trial analysis which controlled for underlying temporal trends (seasonal and secular) in sales and dietary weight, as per protocol with some deviations outlined previously [11]. The revised daily diet per capita, for each intervention, during the discount period and the follow-up period, in absolute weight (gm), energy (MJ) and sodium (mg) for each food and beverage category and total, were calculated by multiplying the mean baseline values by their respective % change (mean and 95%CI) using Microsoft Excel 2013.

#### Intermediate outcome-BMI

BMI change was derived by multiplying the adult and child coefficients of Swinburn et al. [13, 14] on observed percent change in dietary energy, weighted by the baseline population distribution of adults and youth, assuming the measured energy changes were sustained for a 12 month period.

#### Final health outcomes -DALYs

The absolute mean and 95% confidence interval (CI) changes in total estimated BMI, dietary sodium and gram of fruit and vegetables were separately entered to a multi-health state lifetable population cohort Markov model to determine adult population level changes in mortality and morbidity [24]. Final health outcomes measured in DALYs applicable to the adult trial population were derived from the model, which was populated with current data specific to the remote or entire Australian Indigenous population. Data included all-cause mortality rates for the whole population by single year as reported in life expectancy tables for 2010–2012 [25], Indigenous incidence and prevalence of preventable diseases (S1 Table) and prevalence of risk factors within the adult remote Indigenous population for BMI, blood pressure and separate fruit and vegetable consumption in servings per day [26]. Background trends in risk factors were estimated from sales data collected in trial communities prior to the start of the trial.

The effect of each strategy on the incidence of a specific disease was quantified by the potential impact fraction (PIF), which is a function of relative risk of disease and risk factor prevalence. The resulting change in prevalence and mortality of each disease (ischaemic heart disease, stroke, hypertensive heart disease, type 2 diabetes and its sequelae, colon cancer) was modelled over the remaining lifetime of the population, taking all other causes of morbidity and mortality into account. Years of life lived by the population were adjusted for disability associated with each disease specific health-state as well as disability related to other causes as a function of age [27]. Given strong evidence [28] that dietary behaviour change is difficult to maintain, the effects on behaviour were assumed to exponentially decay at a rate of 50% per annum, meaning little effect was sustained beyond five years. Underlying trends in energy and sodium intake identified in the trial statistical analysis (separate from the discount impact) were also assumed to decay at the same rate (50%).

### Costs of intervention

Opportunity costs associated with the strategies were prospectively measured or estimated. Opportunity costs associated with discount promotion design and implementation, design and implementation of the consumer education strategy were sourced from actual invoices received by the trial project manager and trial-based records of participants attending educational activities (i.e. cooking demonstrations and competitions). No extra cost was allocated for store manager time engaged in changing prices, or refreshing individual product tickets as these were considered routine store manager tasks. We assumed that the costs associated with the design phase of the discount and consumer education strategies were likely to be annually recurring to maintain population interest and awareness of discount and consumer education and have included them in the intervention cost. See Tables 1 and 2 for detailed component costing of the strategies.

**Table 1:** Sources of data, cost calculations, and assumptions for each item contributing to the discount promotion cost (AUD 2012–14).

Item	Source	AUD 2012–14	Hours	Comment
**Graphic designer costs of discount promotion banners, fridge stickers and product talkers.**	Actual Invoices	$2,068		Unknown how frequently designs would need changing to serve their purpose. Assumed annually.
**Printing and distribution costs (freight or express post or hand delivery) of branded coloured template discount promotional materials.**	Actual Invoices.	$9,641		Likely to repeat every 6 months.
**Design coordination time costs.**	Project Manager (PM).	$7,434	125	2 days/week for 2 months.
**Discount reimbursement to stores.**	Invoices based on actual sales data.	$221,111		6 months in 20 stores spread over June 2013 to end June 2014.
**Trial staff time costs doing discount promotion in communities over the three phases (pre, during and post discount).**	Time sheets completed associated with travel to communities and pay scales for multiple trial staff.	$17,956	115	Proportion of staff hours allocated to discount promotion at relevant 2013 and 2014 pay scales assuming 46 weeks/year, 5 days/week and 7.8 hours/day. Some worked weekend days used for travel counted as paid in lieu.
**Travel and accommodation to communities for discount promotion.**	Invoices and receipts claimed.	$10,627		All forms of travel used during Oct 2012 to Jul 2014 allocated on basis of staff hours conducting discount promotion.
**Staff time costs of coordination, monitoring, evaluation and support.**	Project Manager (PM) and trial Chief Investigator (CI), pay scales-2013 and 2014.	$50,658	842	PM for 2 days/week for 6 months in each of 2013 and 2014 plus 1 day/month of CI for 6 months in each of 2013 and 2014 covering the discount period (June 13 to June 14).
**Store manager time (additional) promoting the discount to customers, attending to and maintaining discount promotional materials i.e. shelf talkers/banners (not prices).**	Estimate of time at average ALPA and OBS Store manager pay scales.	$38,779	960	Estimated 2 hours/week for 24 weeks in each of 20 stores. Does not include routine weekly changing of prices.
**Total discount strategy costs.**		**$358,275**	**2042**	

**Table 2:** Sources of data, cost calculations, and assumptions for each item contributing to the in-store consumer education program cost (AUD 2012–14).

Item	Source	AUD 2012–14	Hours	Comment
**Graphic designer costs of educational materials and emblazoned T shirts by school children in each of 10 communities.**	Actual Invoices.	$20,579		Assumed an annual cost.
**Printing and distribution costs (freight or express post or hand delivery) of trial branded coloured template consumer educational materials.**	Actual Invoices.	$12,323		Likely to repeat every 6 months.
**Coordination time costs.**	Research officer (RO) and Chief Investigator (CI) staff pay scales.	$34,084	515	2 days/week for 12 months (RO) and 1 day/week for 3 months in 2013 (CI).
**Ongoing recruitment and training by trial staff, of consumer education providers, as indigenous community coordinators, onsite in communities.**	Time sheets completed by trial staff following the travel to communities and pay scales for multiple trial staff involved.	$37,061	330	Hours of each staff member allocated to consumer education multiplied by relevant 2013 and 2014 pay rates with 46 weeks/year, 5 days/week and 7.8 hours/day. Some worked weekend days used for travel are counted as paid in lieu.
**Travel and accommodation for recruiter/trainers.**	Actual Invoices and receipts.	$22,764		All forms of travel used during Oct 2012 to Jul 2014 allocated on basis of the proportion of staff hours conducting recruitment and training of community coordinators in consumer education.
**Materials for educational activities and prizes offered for competitions related to consumer education.**	Actual Invoices and receipts.	$15,532		Prizes comprised fridges, cookers and platters of fruit.
**Community coordinators and Public Health Nutritionist (PHN) time cost conducting consumer education, demonstrations and taste tests.**	Time sheets and individual Invoices reimbursing community coordinators at casual pay rates. PHN assumed employed at mid P2 Professional level of NT government public service [29].	$13,363	341	Proportion of the total cost of community coordinators conducting only consumer education activities plus estimated time of relevant PHNs where required and available.
**Time of community members associated with receiving and participating in the consumer education activities.**	Time estimate is based on number of participants attending taste testings (10 mins) cooking demonstrations, (15 mins) and completing activity sheets (5–7 mins) each. Reported by community coordinators. Assuming all participants were adults using own leisure time.	$1,048	171	A total of 171 hours valued at $6.13/hour. Cost is derived from a weighted leisure time pay rate, i.e. one third of the average weekly earnings rate [30] as at Nov 2012 and one third of the average weekly welfare benefit payments for each adult in a typical family, [31] weighted by the proportion of the trial population working (46%) and on benefits (54%), respectively [32].
**Trial staff time costs undertaking coordination, monitoring, evaluation and support as required.**	Trial staff pay scales–Research Officer (RO) and Project Manager (PM).	$78,803	1225	2 days/week of RO and 1 day/week of PM for the 6 months in each of 2013 and 2014.
**Store manager time supporting the consumer education strategy with prize giving, activity sheets and in store activities.**	Estimate at average of hourly rates of Outback Stores (OBS) and Arnhem Land Progress Aboriginal Corporation (ALPA) store manager salaries.	$9,695	240	1 hour/week for 24 weeks in each of 10 stores.
**Total consumer education strategy costs.**		**$245,252**	**2,821**	

### Cost-offsets

The health sector treatment costs and future potential cost-offsets were estimated by the Markov modelling arising from potential reductions/increases in future lifetime cases of risk factor-related illness, due to the dietary changes following the discount and education strategies in the trial population. The cost to the health sector were derived from estimates of hospital and out of hospital costs associated with prevalent cases of ischaemic heart disease, stroke, hypertensive heart disease, type 2 diabetes and its sequelae, and incident cases of colon cancer in 2011, adjusted by 119% for the additional costs of treating Indigenous Australians [33]. (S1 Table) Net costs equalled the intervention delivery costs plus additional treatment costs, less any cost-offsets.

Intervention costs expended over several years (2012–2014) were deflated using the Australian Consumer Price Index deflator (Dec 2013-Dec 2011)/ Dec 2013 calculated as (104.8–99.8)/104.8 = 4.7% [34]. The net costs were expressed in 2011 Australian dollars (AUD) to be consistent with the year of baseline DALYs, population life expectancy estimates (life tables) and published relative risks of the risk factors.

### Baseline

#### Diet

Baseline dietary weight (gm), energy (MJ) and sodium (mg) for 26 food and beverage categories and total daily intake per capita were based on actual store purchases of food and beverages during the 49-week period, prior to the trial [11].

#### Anthropometry

Baseline BMI of the trial population was calculated based on measured adult height and weight data collected in 2012/13, as part of the Australian Aboriginal and Torres Strait Islander Health Survey [35, 36], reported for remote and very remote geographic areas (combined) by age and gender.

### Discounting

Standard 3% discounting was applied to both costs, cost-offsets and outcomes [37].

### Incremental cost-effectiveness ratios

Incremental cost-effectiveness ratios were reported as net cost/DALY and compared to the commonly accepted threshold for cost-effectiveness in Australia of $50,000 per DALY [38].

### Uncertainty analyses

We used Monte Carlo simulation (2000 iterations) to determine the uncertainty in model outputs based on uncertainty in model inputs. Briggs et al. [39] conducted a comparison of six parametric and non-parametric methods for estimating confidence intervals in Monte Carlo simulation of cost-effectiveness, finding that no one method dominated others under a variety of assumptions. Therefore, we used the Ersatz software [40] (a bootstrap add-in for Microsoft Excel) to calculate 95% uncertainty intervals (mean, 2.5 and 97.5 percentiles) around costs, intermediate dietary outcomes and final health outcome point estimates.

### Trial registration

Australian New Zealand Clinical Trials Registry ACTRN12613000694718. Ethics approval was provided by the combined Northern Territory Department of Health and Menzies School of Health Research Human Research Ethics Committee (HREC-2012-1711), the Central Australian Human Research Ethics Committee (HREC-2012-13 HREC-2012-1711) and Deakin University Human Research Ethics Committee (HREC-2012-243 HREC-2012-1711).

## Results

### Benefits -

#### Diet weight

Modest statistically significant additional per day per person grams of discounted fruit (7gm and 8 gm) and vegetables (5gm and 13gm) were consumed during the discount period under both interventions. Increases in fruit and vegetables were maintained at follow-up in the discount intervention, while increases in vegetable consumption only were maintained by the combined intervention [11]. Consumption of other non-discounted foods also increased (sometimes significantly) leading to an increase in total diet weight (gm) in both interventions (88 (-2.8, 185.4) and 161 (16.6, 320.8)) during the discount period, with wide confidence intervals. At follow-up, the changes in weight of the total diet were 127gm (-13.9, 282.8) and 128gm (-27.9, 303.9) (Table 3).

**Table 3:** Changes from baseline diet in weight, energy and sodium by individual food category, intervention and trial period, (mean and 95% confidence intervals).

	Weight of food and beverages					Energy of food and beverages					Sodium in food and beverages				
	Absolute change (mg/person/day)					Absolute change (MJ/person/day)					Absolute change (mg/person/day)				
**Strategy**		Price discount only		Price discount +education			Price discount only		Price discount +education			Price discount only		Price discount +education	
	Period	Mean	95% CI	Mean	95% CI	Period	Mean	95% CI	Mean	95% CI	Period	Mean	95% CI	Mean	95% CI
**Total baseline diet**	**1360.7**					**8.5**					**2623.5**				
	**During v pre**	88.2	-2.8, 185.4	**161.1**	**16. 6, 320.8**		**0.6**	**0.0,** **1.2**	**0.9**	**0.1,** **1.9**		**216.7**	**13.9, 435.1**	264.3	-19.8, 579.3
	**Post v pre**	127.0	-14.0, 282.8	128.8	-28.0, 303.9		**1.2**	**0.3,** **2.2**	**1.2**	**0.2,** **2.3**		**362.5**	**46.0, 716.4**	232.6	-87.0, 592.4
**All other foods**	**94.1**					**1.3**					**1114.6**				
	**During v pre**	8.0	-0.4, 17.2	4.9	-7.4, 19.0		**0.1**	**0.0,** **0.3**	0.1	-0.1, 0.3		**141.0**	**25.3, 268.5**	116.2	-30.0, 282.1
	**Post v pre**	**17.4**	**3.6, 33.2**	**15.8**	**0.7, 33.3**		**0.3**	**0.1,** **0.5**	**0.3**	**0.0,** **0.5**		158.9	-12.5, 356.9	91.7	-75.3, 285.6
**All other foods good**	**57.3**					**0.5**					**128.4**				
	**During v pre**	1.6	-2.7, 6.3	**7.1**	**0.2, 14.8**		0.0	-0.0, 0.1	**0.1**	**0.0,** **0.1**		0.3	-9.8, 11.3	**18.9**	**1.8, 38.2**
	**Post v pre**	3.9	-2.8, 11.4	6.6	-1.1, 15.2		0.0	-0.0, 0.1	**0.1**	**0.0,** **0.2**		6.4	-9.5, 24.5	16.0	-2.6, 37.4
**Beef**	**27.2**					**0.2**					**23.3**				
	**During v pre**	-0.3	-4.0, 4.0	5.1	-1.6, 13.4		0.0	-0.0, 0.0	0.0	-0.0, 0.1		-0.6	-4.3, 3.7	4.7	-1.7, 12.9
	**Post v pre**	-0.5	-5.9, 6.3	-0.2	-6.3, 7.7		0.0	-0.0, 0.1	0.0	-0.1, 0.1		-0.1	-5.5, 7.0	0.8	-5.3, 8.8
**Breads rolls**	**113.3**					**1.2**					**494.0**				
	**During v pre**	6.3	-1.5, 14.6	12.3	-0.1, 25.9		0.1	-0.0, 0.2	**0.1**	**0.0, 0.3**		26.6	-8.2, 63.9	46.0	-10.1, 108.6
	**Post v pre**	9.9	-2.2, 23.3	9.9	-3.5, 25.0		0.1	-0.0, 0.2	0.1	-0.0, 0.3		43.0	-11.3, 103.4	38.3	-22.7, 107.2
**Cereals**	**84.0**					**1.3**					**148.9**				
	**During v pre**	**9.3**	**1.5, 17.7**	**13.0**	**2.2, 25.1**		**0.1**	**0.0, 0.3**	**0.2**	**0.0, 0.39**		**17.7**	**2.0, 35.1**	**24.5**	**1.7, 50.7**
	**Post v pre**	**15.66**	**3.49, 29.5**	**18.8**	**5.7, 33.9**		**0.2**	**0.1, 0. 5**	**0.3**	**0.1, 0.5**		20.5	-3.2, 48.0	24.7	-1.1, 54.9
**Dairy not milk**	**6.1**					**0.1**					**38.6**				
	**During v pre**	0.6	-0.2, 1.5	**1.9**	**0.5, 3.6**		**0.0**	**0.0, 0.0**	**0.0**	**0.0, 0.0**		3.7	-1.0, 8.9	**7.1**	**0.4, 14.9**
	**Post v pre**	1.0	-0.3, 2.6	**1.9**	**0.4, 3.8**		**0.0**	**0.0, 0.0**	**0.0**	**0.0, 0.0**		5.9	-1.3, 14.5	6.1	-1.5, 15.1
**Diet drinks**	**70.3**					**0.0**					**8.2**				
	**During v pre**	4.3	-3.3, 12.7	5.0	-6.2, 18.1		**0.0**	**0.0, 0.0**	0.0	-0.0, 0.0		0.5	-0.4, 1.5	0.6	-0.7, 2.1
	**Post v pre**	-0.6	-11.1, 11.9	2.6	-9.5, 16.9		0.0	-0.0, 0.0	0.0	-0.0, 0.0		-0.0	-1.3, 1.4	0.3	-1.1, 2.0
**Fruit fresh**	**36.2**					**0.1**					**0.7**				
	**During v pre**	**6.9**	**1.9, 12.5**	**8.2**	**1.1, 16.6**		**0.0**	**0.0, 0.0**	**0.0**	**0.0, 0.0**		0.1	-0.0, 8.9	0.2	-0.0, 0.3
	**Post v pre**	**9.9**	**2.1, 19.3**	4.5	-2.8, 13.4		**0.0**	**0.0, 0.1**	0.0	-0.0, 0.0		0.1	-0.0, 0.3	0.0	-0.1, 0.2
**Fruit other**	**3.9**					**0.0**					**0.3**				
	**During v pre**	**1.0**	**0.1, 2.2**	1.2	-0.2, 3.0		0.0	0.0, 0.0	**0.0**	**0.0, 0.0**		0.0	-0.1, 0.1	0.1	-0.0, 0.3
	**Post v pre**	0.8	-0.5, 2.7	1.1	-0.4, 3.2		0.0	0.0, 0.0	**0.0**	**0.0, 0.0**		-0.0	-0.1, 0.1	0.1	-0.1, 0.3
**Lamb**	**12.5**					**0.1**					**6.9**				
	**During v pre**	2.0	-1.0, 5.9	2.1	-2.5, 8.8		0.0	-0.0, 0.1	0.0	-0.0, 0.1		1.1	-0.6, 3.2	1.2	-1.4, 4.9
	**Post v pre**	2.2	-2.3, 8.7	2.1	-2.9, 9.7		0.0	-0.0, 0.1	0.0	-0.0, 0.1		1.2	-1.3, 4.7	1.2	-1.6, 5.4
**Margarine**	**13.9**					**0.3**					**72.6**				
	**During v pre**	0.5	-0.5, 1.6	0.3	-1.1, 1.9		0.0	-0.0, 0.0	0.0	-0.0, 0.1		0.8	-4.9, 6.9	1.6	-6.5, 10.7
	**Post v pre**	1.4	-0.3, 3.2	1.7	-0.0, 3.7		0.0	-0.0, 0.1	**0.0**	**0.0, 0.1**		4.0	-4.8, 13.9	7.9	-2.0, 19.2
**Milk**	**54.0**					**0.6**					**92.3**				
	**During v pre**	1.9	-1.8, 5.9	3.2	-2.2, 9.1		0.0	-0.0, 0.1	0.1	-0.0, 0.1		5.9	-0.5, 12.8	8.3	-1.0, 18.5
	**Post v pre**	4.9	-1.0, 11.4	5.8	-0.5, 13.0		0.1	-0.0, 0.1	**0.1**	**0.0, 0.2**		8.7	-1.2, 19.6	10.5	-0.2, 22.5
**Other drinks**	**156.3**					**0.4**					**20.9**				
	**During v pre**	7.4	-6.9, 23.1	**24.1**	**0.1, 51.8**		0.0	-0.0, 0.1	**0.1**	**0.0, 0.2**		1.7	-0.3, 4.0	**3.5**	**0.1, 7.5**
	**Post v pre**	9.5	-12.3, 34.6	11.3	-13.3, 40.1		0.0	-0.0, 0.1	0.0	-0.1, 0.1		2.3	-0.9, 5.9	1.9	-1.7, 6.0
**Other meats**	**48.5**					**0.4**					**328.0**				
	**During v pre**	0.8	-4.2, 6.3	1.5	-6.8, 11.4		0.0	-0.0, 0.1	0.0	-0.1, 0.1		0.5	-31.1, 35.5	19.3	-33.7, 82.0
	**Post v pre**	2.7	-5.1, 11.9	-2.1	-10.4, 8.1		0.0	-0.0, 0.1	-0.0	-0.1, 0.1		12.7	-36.6, 70.4	3.2	-52.1, 69.6
**Pork**	**0.8**					**0.0**					**0.6**				
	**During v pre**	-0.0	-0.3, 0.4	0.1	-0.3, 0.8		0.0	0.0, 0.0	0.0	0.0, 0.0		0.1	-0.2, 0.5	0.1	-0.3, 0.8
	**Post v pre**	-0.1	-0.5, 0.5	-0.1	-0.4, 0.6		0.0	0.0, 0.0	0.0	0.0, 0.0		0.1	-0.3, 0.9	0.0	-0.3, 0.9
**Poultry**	**29.2**					**0.2**					**33.6**				
	**During v pre**	1.6	-2.2, 5.9	3.8	-1.9, 10.7		0.0	-0.0, 0.1	0.0	-0.0, 0.1		2.0	-4.2, 9.6	2.8	-6.2, 14.9
	**Post v pre**	4.5	-1.6, 12.0	**7.9**	**0.8, 16.8**		0.0	-0.0, 0.1	**0.1**	**0.0, 0.1**		10.7	-0.6, 25.8	7.6	-3.7, 23.1
**Soft drinks**	**357.0**					**0.6**					**56.2**				
	**During v pre**	19.5	-10.9, 52.6	**48.5**	**0.3, 103.1**		0.0	-0.0, 0.1	**0.1**	**0.0, 0.2**		2.8	-2.2, 8.3	4.1	-3.4, 12.8
	**Post v pre**	17.3	-27.9, 68.8	26.8	-23.9, 85.1		0.0	-0.0, 0.1	0.1	-0.0, 0.1		3.0	-4.5, 11.6	0.6	-7.3, 9.7
**Sugar**	**80.0**					**1.1**					**4.2**				
	**During v pre**	0.9	-5.9, 8.3	6.1	-5.1, 18.9		0.1	-0.0, 0.1	**0.1**	**0.0, 0.3**		0.1	-0.4, 0.7	0.2	-0.5, 1.1
	**Post v pre**	1.8	-8.5, 13.5	1.8	-9.8, 15.3		0.1	-0.0, 0.2	0.1	-0.0, 0.3		0.1	-0.6, 0.9	0.4	-0.4, 1.4
**Tea/coffee**	**7.6**					**0.0**					**0.4**				
	**During v pre**	0.4	-0.2, 1.1	**1.0**	**0.1, 2.1**		0.0	0.0, 0.0	0.0	0.0, 0.0		0.1	-0.0, 0.2	0.0	-0.1, 0.2
	**Post v pre**	0.6	-0.3, 1.7	**1.1**	**0.1, 2.3**		0.0	0.0, 0.0	0.0	0.0, 0.0		0.0	-0.1, 0.2	-0.0	-0.1, 0.1
**Vegetables fresh+frozen**	**50.7**					**0.1**					**7.9**				
	**During v pre**	**4.5**	**0.5, 8.9**	**13.4**	**7.1, 20.4**		**0.0**	**0.0, 0.0**	**0.0**	**0.0, 0.0**		0.4	-0.4, 1.3	**2.4**	**1.0, 3.9**
	**Post v pre**	**8.2**	**1.9, 15.4**	**14.6**	**7.2, 22.9**		**0.0**	**0.0, 0.0**	**0.0**	**0.0, 0.0**		1.1	-0.2, 2.6	**2.0**	**0.5, 3.7**
**Vegetables other**	**14.4**					**0.1**					**42.4**				
	**During v pre**	**2.0**	**0.4, 3.8**	1.3	-1.0, 4.0		**0.0**	**0.0, 0.0**	0.0	0.0, 0.0		**5.2**	**0.5, 10.5**	6.0	-0.8, 13.8
	**Post v pre**	2.0	-0.4, 4.8	1.3	-1.2, 4.3		**0.0**	**0.0, 0.0**	0.0	0.0, 0.0		7.0	-0.3, 15.5	4.8	-2.6, 13.5
**Water**	**43.7**					**0.0**					**0.6**				
	**During v pre**	**7.5**	**0.3, 15.8**	**12.7**	**1.5, 26.6**		0.0	0.0, 0.0	0.0	0.0, 0.0		0.1	-0.0, 0.2	**0.2**	**0.0, 0.4**
	**Post v pre**	**5.1**	**-4.9, 17.8**	**2.3**	**-7.9, 15.4**		**0.0**	**0.0, 0.0**	**0.0**	**0.0, 0.0**		**0.1**	**-0.1, 0.3**	**0.0**	**-0.1, 0.2**

Food and beverages are listed in alphabetical order. Values are rounded to one decimal place. Significant values in bold.

Table 4 shows that price elasticities for all 26 food and beverage categories in response to the concurrent application of the 20% price discount, appeared positive except for beef and pork products, and most were small or inelastic (<1). The statistically significant elasticities included the cross-price elasticity for cereals and the own-price elasticity of the concurrently discounted products.

**Table 4:** Price elasticity of individual and grouped food and beverage categories.

Categories	20% price fall led to these increases in quantity (gm) demanded	1% price fall led to these increases in quantity (gm) demanded	95%CI
**Total diet**	6%	0.32%	-0.01%, 0.68%
**Grouped food /beverage categories**			
**All Fruit+Vegetables**	**13%**	**0.63%**	**0.21%, 1.08%**
**Discounted drinks**	**11%**	**0.54%**	**0.00%, 1.15%**
**All other food lines**	5%	0.27%	-0.06%, 0.62%
**Sweet drinks**	5%	0.25%	-0.14%, 0.68%
**Individual food/beverage categories**			
**All other foods**	9%	0.43%	-0.02%, 0.92%
**All other foods good**	3%	0.14%	-0.23%, 0.55%
**Beef**	-1%	-0.05%	-0.73%, 0.73%
**Breads rolls**	6%	0.28%	-0.07%, 0.64%
**Cereals**	**11%**	**0.55%**	**0.09%, 1.05%**
**Dairy not milk**	10%	0.48%	-0.19%, 1.25%
**Diet drinks**	6%	0.30%	-0.23%, 0.90%
**Fruit fresh**	**19%**	**0.95%**	**0.26%, 1.72%**
**Fruit other**	**26%**	**1.31%**	**0.07%, 2.85%**
**Lamb**	16%	0.81%	-0.41%, 2.36%
**Margarine**	4%	0.19%	-0.18%, 0.59%
**Milk**	4%	0.18%	-0.17%, 0.55%
**Other drinks**	5%	0.24%	-0.22%, 0.74%
**Other meats**	2%	0.08%	-0.43%, 0.65%
**Pork**	-6%	-0.29%	-2.01%, 2.43%
**Poultry**	5%	0.27%	-0.37%, 1.01%
**Soft drinks**	5%	0.27%	-0.15%, 0.74%
**Sugar**	1%	0.06%	-0.37%, 0.52%
**Tea coffee**	5%	0.26%	-0.15%, 0.70%
**Vegetables fresh+frozen**	**9%**	**0.44%**	**0.05%, 0.87%**
**Vegetables other**	**14%**	**0.69%**	**0.13%, 1.32%**
**Water**	**17%**	**0.85%**	**0.03%, 1.81%**

Food and beverages are listed in alphabetical order.

Statistically significant values are highlighted.

#### Diet energy

The changes in composition and weight of the total diet (Table 3) were accompanied by statistically significant increases and wide confidence intervals in total daily dietary energy per capita in both interventions during the discount period and at follow-up. Initially during the discount period the absolute increase was 0.57 MJ (0.01 to 1.18) in the discount intervention and 0.92 MJ (0.05 to 1.87) in the combined intervention. At follow-up, the statistically significant additional per capita daily dietary energy was nearly identical at 1.18 MJ (0.27, 2.18) and 1.18 MJ (0.19, 2.29) in the respective interventions. While the discount only intervention led to unexpected significant extra energy from cereals, “all other foods” (e.g. pizza, hamburgers, snack foods, confectionary) and dairy products, the combined intervention led to unexpected significant extra energy from a wider range of food and beverage categories during the discount period and/or follow up. Additional energy sources included bread, “all other foods good” (e.g. eggs, fish, nuts, seeds), fruit other, poultry, sugar, soft drinks and other drinks, as well as the same three categories identified in the discount only intervention.

#### Diet sodium

During the discount period, the additional daily per capita sodium intake was 216.67 mg (13.86 to 435.07) in the discount only intervention, sourced from “all other foods”, cereals and tinned/ dried vegetables, while the change was 264.26 mg (-19.80 to 579.30) in the combined intervention, arising from cereals, “all other foods good”, dairy, other drinks and discounted vegetables. At follow-up, the additional daily per capita sodium intake was 362.46mg (46.05 to 716.38) in the discount only intervention, and 232.55 mg (-19.80 to 579.30) in the combined intervention. No substitution effects away from soft drinks or unhealthy foods were measured (Table 3).

#### BMI change

Both interventions resulted in significantly increased BMI based on the daily per capita increases of dietary energy measured during the discount and at follow-up, compared to baseline. At the end of the discount period, the discount only intervention resulted in an increased BMI of 1.15 (0.28, 2.67) units, while the combined intervention was associated with an increase of 1.89 (0.49, 4.21) units. At follow-up, the discount only intervention was associated with an increased BMI of 2.38 (0.81, 4.62) units, and the combined intervention 2.37 (0.78, 4.75) units.

#### DALYs

Over the lifetime of the trial based population, additional cases of risk factor (excess BMI and higher sodium) related diseases, (IHD, diabetes, stroke) could be expected to lead to additional fatal and non-fatal DALY outcomes in spite of the modest increase in fruit and vegetable consumption. Additional DALYs would be lost (reported as negative) due to the increased BMI and sodium consumption; in the discount period -21 DALYs (-28 to -15) and -36 DALYs (-47 to -25) and at follow-up, -48 DALYs (-60 to -36) and -45 DALYs (-58 to -34). There was considerable uncertainty surrounding the DALY estimates.

#### Costs

The total cost of the 24-week discount was AUD200,000 (i.e. average $10,000 per store). The total cost of the consumer education component of the combined intervention was AUD170,000 (i.e. average $17,000 per store). The detailed costs in AUD 2012–2014 of each strategy is provided in Tables 1 and 2 and summarised by trial phase, strategy and community in Table 5.

**Table 5:** Total cost by intervention group and trial phase. (AUD 2012–14).

	Discount only communities (n = 10)			Combined discount plus consumer education communities (n = 10)		
Cost type	Design	Implementation	Total	Design	Implementation	Total
**Discount**	$9,572	$205,346	$214,918	$9,572	$133,785	$143,357
**Consumer education**	0	0	0	$66,986	$178,266	$245,252
**Total costs**	$9,572	$205,346	$214,918	$76,558	$312,051	$388,609
**Average cost per store**			$21,492			$38,861

There were no cost offsets from reduced cases of diet-related diseases, (over the lifetime of the population), hence net costs to the health sector of the 24-week discount intervention was an additional AUD500,000 ($410,000 to $590,000), while for the combined intervention it was AUD860,000 ($710,000 to $1million). At follow-up, net costs of each strategy were greater due to more estimated future cases of disease. (Table 6).

**Table 6:** DALYs, costs, cost offsets, net costs and ICERS (AUD2011).

Strategies Compared to pre discount period baseline	DALYs Mean (95% UI)	Intervention costs AUD ‘000	Cost Offsets AUD ‘000 Mean (95% UI)	Net Costs AUD ‘000 Mean (95% UI)	ICER AUD ‘000 Mean(95%UI)
**Discount only during 24-week discount period**	**-21(-28 to -15)**	**200**	**290 (210 to 390)**	**500 (410 to 590)**	**Dominated**
**Discount+consumer education during 24-week discount period**	**-36 (-47 to -25)**	**370**	**490 (340 to 640)**	**860 (710 to 1,000)**	**Dominated**
**Discount only at follow-up**	**-48 (-60 to -36)**	**200**	**640 (480 to 830)**	**850 (690 to 1,000)**	**Dominated**
**Discount+consumer education at follow-up**	**-45 (-58 to -34)**	**370**	**610 (450 to 790)**	**980 (820 to 1,200)**	**Dominated**

### Incremental cost effectiveness

**Fig 1:**
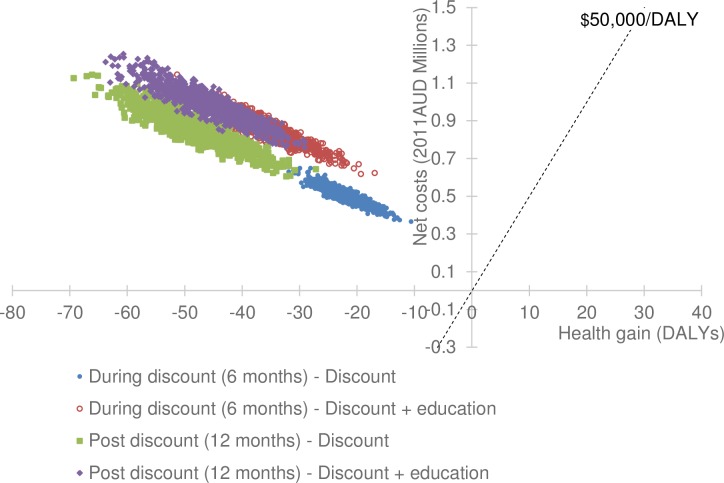
Cost-effectiveness plane.

Compared to current practice, a 20% discount on fruit, vegetables, artificially sweetened soft drinks and bottled water, with or without consumer education, cost more money without leading to health gain; this is described as being dominated and offering poor value for money (Table 6). A cost-effectiveness plane (Fig 1) presents visual representation of all the thousands of results determined by the modelling as uncertainty is incorporated. It displays on the horizontal axis the changes in estimated health outcome and on the vertical axis the concurrent change in costs when the change in outcomes occur. All of the iterations of the model estimated health losses would occur while costs increased when compared with doing no discounts or consumer education.

## Discussion

Indigenous consumers in remote communities responded to 20% discounted prices with considerable variability. The own price elasticity of fruit and vegetables was statistically significant, in the hypothesised positive direction and consistent with previous studies. While there were modest and lasting increases in consumption of healthy fruit and vegetables in this population, there was an accompanying increase in consumption of other foods, notably cereals (cross price elasticity 0.55: 95%CI 0.09%, 1.05%) which led to increased total dietary energy and sodium intake, both of which have been linked to poorer lifetime health outcomes. Since the negative changes to population health as modelled, occurred in conjunction with additional costs to both the health and retail sectors, the 20% discounts with or without consumer education interventions offer poor value for money and should not be recommended. While we are aware that, on equity grounds a threshold of cost-effectiveness, higher than $50,000 per DALY, could be adopted in such a population experiencing disadvantage [33], our cost-effectiveness results fall completely in the top left quadrant of the cost-effectiveness plane (Fig 1).

There may have been non-health benefits that occurred during the trial such as improvements in food security and self-efficacy, which were not able to be separately captured in this quantitative economic analysis. All store boards invited to participate in the study agreed to and hence supported a discount on fruit and vegetables, although during the consultation phase of this study recommended that the discount be applied across a wider range of healthier products and were concerned about the potential limited effect of applying a discount to fruit and vegetable only.

A recent publication [41] found that 76% of the trial population customers surveyed, reported food insecurity in the last 12 months, with 40% of these respondents reporting to run out of food and not be able to afford more, once per week. The 20% discount was also perceived by consumers as a positive price reduction on fruit and vegetables.

### Comparison with the literature

#### Trials

No published trials have studied the whole diet or analysed impact on health (measured as DALYs) and costs in this remote Indigenous population [10], so no direct comparisons of our cost-effectiveness results are possible.

Other trials of fiscal interventions conducted in populations experiencing disadvantage [42] have mixed results while they offered different sized discounts, used different methods of redeeming the discounts and importantly did not capture the impact on the whole trial population diet.

#### Modelling studies

Two previously modelled economic evaluations of food voucher interventions for low income groups (Alston et al. 2009 [43] and de Mouzon et al. 2012 [44]) suggested there may be unintended consequences on the quantity of other food lines consumed due to underlying consumer preferences and potential market forces of supply and demand. Their concerns are borne out by our trial-based study.

Previous modelling work in the Australian Indigenous population [8], which assumed the weight of the diet would remain constant, while discounted foods were substituted for other less healthy options, can be compared to this SHOP@RIC trial. The important dietary weight maintenance assumption of that earlier modelled study which found discount strategies were promising, has been found not to apply in this trial population. Instead the measured weight, energy and sodium of the total diet increased due to additional consumption of a relatively important dietary component (cereals) previously reported to be consumed in situations where people have less money for food, [45] following introduction of price discounts on selected items. In such a population experiencing food insecurity, it is not surprising that with more dollars available from the discount, cheaper and sustaining cereal calories were purchased [46].

#### Price elasticity studies

We are further supported in our findings by Ni Murchu et al. [9] where similarly important for health, cross-price elasticities of fruit and vegetables with cakes/biscuits and ready to eat foods were found in the New Zealand population, meaning that a price fall in fruit and vegetables would be associated with an increase in purchases of both groups of products. A second recent modelling study for the entire Australian population [24], found cross price elasticity effects similar to ours, which led to population health losses when discounts on fruits and vegetables were offered in isolation, again due to potential increased consumption of foods high in sodium and energy.

### Strengths

We were able to consider these multiple dietary risk factors (low fruit and vegetables, high sodium and energy) given the availability of recently measured data on risk factor prevalence in the remote Australian Indigenous population [35, 36], and up to date evidence linking dietary risk factors to health outcomes (diabetes, heart conditions, cancers and stroke). Also, actual store sales data enabled dietary changes and population responsiveness to price changes, to be analysed in considerable detail and be more objectively determined than relying on participant recall.

Given the robust stepped-wedge trial design and statistical methods, we were able to isolate dietary change following the price discount intervention with and without consumer education.

This is the first time that responsiveness to a specified concurrent set of food price changes has been examined in remote Indigenous communities, and estimated, within a total diet, incorporating an assessment of own-price and cross-price elasticities for 26 food and beverage categories of interest to nutritionists, policy makers and economists alike. While it was not possible to employ standard regression techniques at a household level, we have estimated for this trial population, their community-level diet impacts with uncertainty represented by confidence intervals.

The costs have been well documented, based largely on objective data (actual Invoices) and clearly identify the complex components of fiscal and consumer education strategies within the context of remote Australian Indigenous communities.

### Limitations

There are some limitations inherent in this analysis which require caution be attached to the results.

Firstly no change from other potential dietary risk factors (i.e. water consumption or fibre, fats, red or processed meats) were assessed or modelled into DALYs, due to the current absence of data on the distribution of the respective dietary parameters in the remote Indigenous population. The combined impact of these exclusions is difficult to estimate since some factors may have contributed large or small health benefits while others may have added to health losses. Future analysis of the actual dietary data from this study would be valuable in this regard.

Secondly we were unable to separately identify child and adult baseline anthropometric measures, diet composition or response to the discounts and consumer education. Therefore we present a whole of population analysis which can only lead to conclusions concerning the whole communities rather than any subgroup within it, while noting the wide confidence intervals in the results.

Thirdly, the trial design and budget precluded the measurement of actual change in adult BMI. We relied instead on baseline height, weight and BMI measurements recently surveyed in the entire remote Indigenous population assuming our trial population is representative of the wider group. Since the measured trial baseline total dietary energy (8530kJ per day per capita) was lower than that recommended (8700) to support daily energy requirements of this adult and child trial population it is evident that the trial stores were not the only source of foods and/or that the trial population was undernourished. This may mean that the measured increase in average population energy and modelled BMI change could be considered beneficial and would not have led to the harm in the whole population as enumerated. Alternatively, population diets may have been supplemented by shopping at other food stores located out of the community or by consuming traditional foods such as fish, kangaroo, berries and yams [47]. Since we believe there is sound reason to consider the store-based purchases a reliable measure of total population diet [48] and have assumed the methodology of Swinburn et al. [13, 14] is sound for converting energy increase to BMI change, these findings can only be improved by expensive and invasive repeated measurement of BMI, supplemented by self-report dietary intake, in any future trials of this nature.

Finally, we have incorporated all of the statistical uncertainty related to the population response to the 20% discount offered (i.e. price elasticities) with and without consumer education, in the calculation of health outcomes. This means that statistically insignificant elasticity changes and dietary changes for any of the 26 food and beverage categories have been modelled to health outcomes and there are wider uncertainty intervals than would have been presented if only the statistically significant changes were incorporated in the modelled DALYs. By examining as many dietary components over the multiple time periods we may have introduced the chance of random statistically significant values.

## Conclusions

Small and complex total dietary changes occur when adopting single focus price discount strategies, which can lead to unintended health consequences for participants. The estimated potential poorer health outcomes were gained at a cost. Compared to the threshold usually adopted in Australia, these strategies *would not be* deemed cost-effective based on evidence that measured increases in sodium, energy and estimated change in BMI were not beneficial at a population level. While offering promise, discounts on targeted healthy food lines coupled with other strategies such as price increases (taxes) on unhealthy food lines, combined with attention to product availability (fewer discretionary foods), product reformulation (to reduce sodium availability) and other marketing strategies, may offer a preferable strategy. Other relevant issues such as non-health benefits (food insecurity), food price equity and store viability which were not separately captured in this quantitative analysis, also need due consideration by policy makers and store committees.

## Supporting information

S1 Table Indigenous input data used to model DALYs and costs averted. (PDF)
