# Supplementary material for: The cost-effectiveness of a 20% price discount on fruit, vegetables, diet drinks and water, trialled in remote Australia to improve Indigenous health
Source: PLoS One. 2018 Sep 27;13(9):e0204005. doi: 10.1371/journal.pone.0204005 (PMC6160015; doi:10.1371/journal.pone.0204005)
Supplement: S1 Table — (PDF) [file pone.0204005.s001.pdf]

	A	B	C	D	E	F	G	H	I	J
1	**DATA INPUTS**									
2			not available	2011 remote	ABS remote groups 2010-12					
3			**Population**		**Mortality**	**Incidence**				
4	sex	age	1-year	5-year	mortality rate	I20-I25: Ischaemic heart	I60-I69: Cerebrovascular	E11,E14: Diabetes	C50: Breast	C18-20: Colorectum
5	male	0			0.01131	0.00000	0.00000	0.000892	0	0
6	male	1			0.00193	0.00000	0.00000	0.000882	0	0
7	male	2		7813	0.00193	0.00000	0.00000	0.000872	0	0
8	male	3			0.00193	0.00000	0.00001	0.000862	0	0
9	male	4			0.00193	0.00000	0.00001	0.000853	0	0
10	male	5			0.00149	0.00000	0.00001	0.000848	0	0
11	male	6			0.00149	0.00000	0.00001	0.000849	0	1.2723E-07
12	male	7		7457	0.00149	0.00000	0.00000	0.000857	0	2.9694E-07
13	male	8			0.00149	0.00000	0.00000	0.000878	0	4.7953E-07
14	male	9			0.00149	0.00000	0.00000	0.000927	0	6.4539E-07
15	male	10			0.00150	0.00000	0.00000	0.001022	0	7.6493E-07
16	male	11			0.00150	0.00000	0.00000	0.001181	0	8.2365E-07
17	male	12		7216	0.00150	0.00001	0.00000	0.001422	0	8.676E-07
18	male	13			0.00150	0.00003	0.00005	0.001758	0	9.5796E-07
19	male	14			0.00150	0.00006	0.00012	0.002184	0	1.1559E-06
20	male	15			0.00786	0.00009	0.00020	0.002688	0	1.5226E-06
21	male	16			0.00786	0.00012	0.00027	0.003261	0	2.0995E-06
22	male	17		6406	0.00786	0.00014	0.00031	0.003891	0	2.8501E-06
23	male	18			0.00786	0.00016	0.00034	0.004572	0	3.7179E-06
24	male	19			0.00786	0.00017	0.00034	0.005306	0	4.6466E-06
25	male	20			0.01294	0.00018	0.00033	0.006101	0	5.5799E-06
26	male	21			0.01294	0.00018	0.00032	0.006963	0	6.4794E-06
27	male	22		6412	0.01294	0.00017	0.00031	0.007898	0	7.3789E-06
28	male	23			0.01294	0.00018	0.00032	0.008913	0	8.33E-06
29	male	24			0.01294	0.00020	0.00034	0.010023	0	9.3844E-06
30	male	25			0.01329	0.00029	0.00035	0.011238	0	1.0594E-05
31	male	26			0.01329	0.00046	0.00035	0.012574	0	1.2031E-05
32	male	27		5539	0.01329	0.00074	0.00031	0.014042	0	1.3851E-05
33	male	28			0.01329	0.00114	0.00025	0.015644	0	1.6231E-05
34	male	29			0.01329	0.00160	0.00018	0.017326	0	1.9347E-05
35	male	30			0.02096	0.00203	0.00014	0.01902	0	2.3376E-05
36	male	31			0.02096	0.00235	0.00018	0.02066	0	2.8376E-05
37	male	32		4758	0.02096	0.00246	0.00031	0.022179	0	3.3937E-05
38	male	33			0.02096	0.00232	0.00058	0.023536	0	3.9532E-05
39	male	34			0.02096	0.00204	0.00092	0.024795	0	4.4632E-05
40	male	35			0.02666	0.00177	0.00129	0.026045	0	4.871E-05
41	male	36			0.02666	0.00167	0.00163	0.027377	0	5.157E-05
42	male	37		4519	0.02666	0.00189	0.00188	0.028881	0	5.4335E-05
43	male	38			0.02666	0.00252	0.00200	0.030611	0	5.8464E-05
44	male	39			0.02666	0.00350	0.00202	0.032479	0	6.5412E-05
45	male	40			0.03837	0.00469	0.00198	0.034364	0	7.6635E-05
46	male	41			0.03837	0.00597	0.00192	0.036141	0	9.3072E-05
47	male	42		4139	0.03837	0.00722	0.00188	0.037689	0	0.00011359
48	male	43			0.03837	0.00834	0.00191	0.038911	0	0.00013655
49	male	44			0.03837	0.00932	0.00196	0.039822	0	0.00016031
50	male	45			0.04737	0.01016	0.00200	0.040462	0	0.0001832
51	male	46			0.04737	0.01088	0.00199	0.040872	0	0.00020445
52	male	47		3538	0.04737	0.01150	0.00188	0.041094	0	0.00022674
53	male	48			0.04737	0.01202	0.00167	0.041173	0	0.00025359
54	male	49			0.04737	0.01250	0.00144	0.041183	0	0.00028854
55	male	50			0.06400	0.01298	0.00132	0.0412	0	0.00033512
56	male	51			0.06400	0.01350	0.00143	0.041303	0	0.00039551
57	male	52		2856	0.06400	0.01411	0.00188	0.04157	0	0.00046647
58	male	53			0.06400	0.01485	0.00276	0.042044	0	0.00054341
59	male	54			0.06400	0.01567	0.00392	0.042632	0	0.00062173

	A	B	C	D	E	F	G	H	I	J
60	male	55			0.08815	0.01653	0.00515	0.043206	0	0.00069684
61	male	56			0.08815	0.01736	0.00626	0.043639	0	0.00076567
62	male	57		2187	0.08815	0.01812	0.00707	0.043804	0	0.00083126
63	male	58			0.08815	0.01878	0.00742	0.043619	0	0.00089816
64	male	59			0.08815	0.01936	0.00743	0.043192	0	0.00097095
65	male	60			0.12003	0.01991	0.00727	0.042677	0	0.00105418
66	male	61			0.12003	0.02050	0.00709	0.04223	0	0.00115115
67	male	62		1384	0.12003	0.02118	0.00707	0.042004	0	0.00126006
68	male	63			0.12003	0.02197	0.00732	0.042096	0	0.00137784
69	male	64			0.12003	0.02283	0.00777	0.042375	0	0.00150144
70	male	65			0.15586	0.02367	0.00829	0.04265	0	0.00162779
71	male	66			0.15586	0.02444	0.00878	0.042732	0	0.00175401
72	male	67		962	0.15586	0.02505	0.00911	0.042431	0	0.00187792
73	male	68			0.15586	0.02546	0.00920	0.04162	0	0.00199754
74	male	69			0.15586	0.02568	0.00912	0.040425	0	0.00211089
75	male	70			0.22841	0.02577	0.00900	0.039033	0	0.00221597
76	male	71			0.22841	0.02576	0.00896	0.037632	0	0.00231153
77	male	72		521	0.22841	0.02569	0.00911	0.036412	0	0.00239924
78	male	73			0.22841	0.02562	0.00953	0.035498	0	0.00248146
79	male	74			0.22841	0.02556	0.01015	0.034774	0	0.0025606
80	male	75			0.28687	0.02553	0.01085	0.034061	0	0.00263904
81	male	76			0.28687	0.02555	0.01151	0.033183	0	0.0027179
82	male	77		483	0.28687	0.02566	0.01202	0.031961	0	0.00279322
83	male	78			0.28687	0.02585	0.01228	0.030289	0	0.00285979
84	male	79			0.28687	0.02610	0.01235	0.02835	0	0.00291236
85	male	80			0.35135	0.02636	0.01228	0.026398	0	0.00294572
86	male	81			0.35135	0.02661	0.01215	0.024688	0	0.00295634
87	male	82			0.35135	0.02679	0.01202	0.023474	0	0.0029474
88	male	83			0.35135	0.02688	0.01193	0.022952	0	0.00292381
89	male	84			0.35135	0.02689	0.01191	0.023087	0	0.00289044
90	male	85			1.000000	0.02684	0.01192	0.023784	0	0.00285218
91	male	86			1.000000	0.02684	0.01192	0.024951	0	0.00285218
92	male	87			1.000000	0.02684	0.01192	0.026493	0	0.00285218
93	male	88			1.000000	0.02684	0.01192	0.028316	0	0.00285218
94	male	89			1.000000	0.02684	0.01192	0.030327	0	0.00285218
95	male	90			1.000000	0.02684	0.01192	0.032432	0	0.00285218
96	male	91			1.000000	0.02684	0.01192	0.032432	0	0.00285218
97	male	92			1.000000	0.02684	0.01192	0.032432	0	0.00285218
98	male	93			1.000000	0.02684	0.01192	0.032432	0	0.00285218
99	male	94			1.000000	0.02684	0.01192	0.032432	0	0.00285218
100	male	95			1.000000	0.02684	0.01192	0.032432	0	0.00285218
101	male	96			1.000000	0.02684	0.01192	0.032432	0	0.00285218
102	male	97			1.000000	0.02684	0.01192	0.032432	0	0.00285218
103	male	98			1.000000	0.02684	0.01192	0.032432	0	0.00285218
104	male	99			1.000000	0.02684	0.01192	0.032432	0	0.00285218
105	male	100			1.000000	0.02684	0.01192	0.032432	0	0.00285218
106			not available	2011 remote	ABS remote groups 2010-12					
107			**Population**		**Mortality**	**Incidence**				
108	sex	age	1-year	5-year	mortality rate	I20-I25: Ischaemic heart diseases	I60-I69: Cerebrovascular diseases	E11,E14: Diabetes	C50: Breast	C18-20: Colorectum
109	female	0			0.00930	0.00000	0.00000	0.00047	0	0
110	female	1			0.00189	0.00001	0.00000	0.000496	0	0
111	female	2		7516	0.00189	0.00000	0.00000	0.000537	0	4.2145E-07
112	female	3			0.00189	0.00000	0.00001	0.0006	0	7.641E-07
113	female	4			0.00189	0.00000	0.00002	0.000673	0	9.4914E-07
114	female	5			0.00125	0.00000	0.00002	0.00074	0	8.9777E-07
115	female	6			0.00125	0.00000	0.00002	0.000784	6.69E-08	5.765E-07
116	female	7		7237	0.00125	0.00003	0.00000	0.000788	1.37E-07	1.3306E-07
117	female	8			0.00125	0.00008	0.00000	0.000745	1.74E-07	0

	A	B	C	D	E	F	G	H	I	J
118	female	9			0.00125	0.00014	0.00000	0.000679	1.4E-07	0
119	female	10			0.00140	0.00020	0.00000	0.000626	0	0
120	female	11			0.00140	0.00025	0.00000	0.00062	0	9.2474E-07
121	female	12		6665	0.00140	0.00026	0.00000	0.000695	0	2.2518E-06
122	female	13			0.00140	0.00024	0.00010	0.000881	0	3.7335E-06
123	female	14			0.00140	0.00022	0.00022	0.001182	0	5.1222E-06
124	female	15			0.00365	0.00024	0.00035	0.001595	0	6.1702E-06
125	female	16			0.00365	0.00033	0.00048	0.002119	9.52E-07	6.6998E-06
126	female	17		5924	0.00365	0.00052	0.00057	0.002753	1.93E-06	6.8119E-06
127	female	18			0.00365	0.00086	0.00061	0.003495	2.44E-06	6.6777E-06
128	female	19			0.00365	0.00128	0.00062	0.004342	1.96E-06	6.468E-06
129	female	20			0.00527	0.00173	0.00060	0.005291	0	6.3538E-06
130	female	21			0.00527	0.00214	0.00058	0.006342	0	6.4807E-06
131	female	22		6169	0.00527	0.00247	0.00057	0.00749	0	6.8921E-06
132	female	23			0.00527	0.00265	0.00058	0.008738	0	7.6065E-06
133	female	24			0.00527	0.00272	0.00059	0.010105	0	8.6421E-06
134	female	25			0.00882	0.00271	0.00061	0.011612	0	1.0017E-05
135	female	26			0.00882	0.00264	0.00061	0.013281	1.41E-05	1.176E-05
136	female	27		5729	0.00882	0.00257	0.00057	0.015136	3.33E-05	1.3944E-05
137	female	28			0.00882	0.00250	0.00049	0.017183	5.56E-05	1.6649E-05
138	female	29			0.00882	0.00245	0.00040	0.019366	7.85E-05	1.996E-05
139	female	30			0.00875	0.00242	0.00036	0.021614	0.0001	2.3958E-05
140	female	31			0.00875	0.00240	0.00040	0.023856	0.000119	2.8672E-05
141	female	32		4996	0.00875	0.00238	0.00057	0.02602	0.000136	3.3913E-05
142	female	33			0.00875	0.00237	0.00089	0.028047	0.000153	3.9439E-05
143	female	34			0.00875	0.00239	0.00131	0.029919	0.000174	4.5006E-05
144	female	35			0.01608	0.00246	0.00177	0.031631	0.0002	5.0372E-05
145	female	36			0.01608	0.00260	0.00218	0.033176	0.000233	5.5505E-05
146	female	37		4692	0.01608	0.00284	0.00249	0.034549	0.000271	6.1215E-05
147	female	38			0.01608	0.00319	0.00265	0.03576	0.000313	6.8522E-05
148	female	39			0.01608	0.00363	0.00267	0.036881	0.000357	7.8446E-05
149	female	40			0.02543	0.00413	0.00262	0.038001	0.0004	9.2007E-05
150	female	41			0.02543	0.00467	0.00254	0.039208	0.000442	0.00010979
151	female	42		4435	0.02543	0.00521	0.00249	0.040592	0.000482	0.00013061
152	female	43			0.02543	0.00574	0.00250	0.042194	0.000522	0.00015287
153	female	44			0.02543	0.00626	0.00255	0.043869	0.000561	0.00017496
154	female	45			0.03117	0.00681	0.00259	0.045425	0.0006	0.00019526
155	female	46			0.03117	0.00740	0.00258	0.046672	0.00064	0.00021274
156	female	47		3612	0.03117	0.00806	0.00249	0.047418	0.00068	0.00022869
157	female	48			0.03117	0.00880	0.00230	0.047543	0.000721	0.00024498
158	female	49			0.03117	0.00958	0.00209	0.047214	0.000761	0.00026348
159	female	50			0.04659	0.01035	0.00198	0.046669	0.0008	0.00028606
160	female	51			0.04659	0.01106	0.00208	0.046146	0.000838	0.0003143
161	female	52		3175	0.04659	0.01167	0.00249	0.045884	0.000876	0.00034861
162	female	53			0.04659	0.01215	0.00329	0.046041	0.000915	0.00038914
163	female	54			0.04659	0.01256	0.00432	0.046463	0.000956	0.000436
164	female	55			0.06651	0.01301	0.00539	0.046916	0.001	0.00048933
165	female	56			0.06651	0.01358	0.00632	0.047165	0.001047	0.000549
166	female	57		2321	0.06651	0.01438	0.00691	0.046977	0.001094	0.00061391
167	female	58			0.06651	0.01545	0.00704	0.046196	0.001138	0.0006827
168	female	59			0.06651	0.01672	0.00688	0.044971	0.001174	0.00075399
169	female	60			0.07709	0.01808	0.00665	0.043531	0.0012	0.00082643
170	female	61			0.07709	0.01941	0.00658	0.042105	0.001213	0.00089883
171	female	62		1557	0.07709	0.02059	0.00691	0.04092	0.001215	0.00097065
172	female	63			0.07709	0.02154	0.00779	0.040149	0.001211	0.00104154
173	female	64			0.07709	0.02224	0.00905	0.03974	0.001204	0.00111112
174	female	65			0.10885	0.02270	0.01043	0.039587	0.0012	0.00117904
175	female	66			0.10885	0.02293	0.01169	0.039582	0.001201	0.00124554
176	female	67		1135	0.10885	0.02294	0.01258	0.039618	0.001204	0.00131325
177	female	68			0.10885	0.02275	0.01292	0.039616	0.001207	0.0013854
178	female	69			0.10885	0.02242	0.01286	0.039603	0.001207	0.00146524
179	female	70			0.18178	0.02202	0.01263	0.039633	0.0012	0.00155599
180	female	71			0.18178	0.02162	0.01246	0.039762	0.001185	0.00165899
181	female	72		788	0.18178	0.02129	0.01258	0.040044	0.001163	0.00176796

	A	B	C	D	E	F	G	H	I	J
182	female	73			0.18178	0.02109	0.01315	0.040491	0.001139	0.00187472
183	female	74			0.18178	0.02104	0.01405	0.04094	0.001117	0.00197109
184	female	75			0.23437	0.02115	0.01510	0.041185	0.0011	0.00204889
185	female	76			0.23437	0.02145	0.01610	0.041019	0.001091	0.00210267
186	female	77		856	0.23437	0.02194	0.01688	0.040237	0.001089	0.00213783
187	female	78			0.23437	0.02263	0.01730	0.038715	0.001091	0.00216252
188	female	79			0.23437	0.02346	0.01740	0.036666	0.001095	0.00218488
189	female	80			0.32848	0.02435	0.01730	0.034387	0.0011	0.00221303
190	female	81			0.32848	0.02525	0.01709	0.032174	0.001103	0.00225331
191	female	82			0.32848	0.02608	0.01688	0.030324	0.001104	0.00230473
192	female	83			0.32848	0.02680	0.01676	0.02907	0.001103	0.00236452
193	female	84			0.32848	0.02740	0.01672	0.028392	0.001102	0.00242989
194	female	85			1.00000	0.02792	0.01674	0.028209	0.0011	0.00249804
195	female	86			1.00000	0.02792	0.01674	0.028438	0.0011	0.00249804
196	female	87			1.00000	0.02792	0.01674	0.028996	0.0011	0.00249804
197	female	88			1.00000	0.02792	0.01674	0.029802	0.0011	0.00249804
198	female	89			1.00000	0.02792	0.01674	0.030772	0.0011	0.00249804
199	female	90			1.00000	0.02792	0.01674	0.031825	0.0011	0.00249804
200	female	91			1.00000	0.02792	0.01674	0.031825	0.0011	0.00249804
201	female	92			1.00000	0.02792	0.01674	0.031825	0.0011	0.00249804
202	female	93			1.00000	0.02792	0.01674	0.031825	0.0011	0.00249804
203	female	94			1.00000	0.02792	0.01674	0.031825	0.0011	0.00249804
204	female	95			1.00000	0.02792	0.01674	0.031825	0.0011	0.00249804
205	female	96			1.00000	0.02792	0.01674	0.031825	0.0011	0.00249804
206	female	97			1.00000	0.02792	0.01674	0.031825	0.0011	0.00249804
207	female	98			1.00000	0.02792	0.01674	0.031825	0.0011	0.00249804
208	female	99			1.00000	0.02792	0.01674	0.031825	0.0011	0.00249804
209	female	100			1.00000	0.02792	0.01674	0.031825	0.0011	0.00249804

	K	L	M	N	O	P	Q	R	S	T
1										
2						From Indigenous BOD (remote)				
3	Case fatality					Prevalence (initial)				
4	I20-I25: Ischaemic heart	I60-I69: Cerebrovascular	E11,E14: Diabetes	C50: Breast	C18-20: Colorectum	I20-I25: Ischaemic heart	I60-I69: Cerebrovascular	E11,E14: Diabetes	C50: Breast	C18-20: Colorectum
5	0.0000	0.0066	0	0	0	0	2.13E-08	0.000196	0	0
6	0.0000	0.0064	4.31E-09	0	0	0	1.29E-08	0.001159	0	0
7	0.0000	0.0057	0	0	0	0	1.29E-08	0.001159	0	0
8	0.0000	0.0050	-1.8E-08	0	0	0	1.29E-08	0.001159	0	0
9	0.0000	0.0043	-4E-08	0	0	0	1.29E-08	0.001159	0	0
10	0.0000	0.0036	-5.3E-08	0	0	0	4.08E-08	0.002844	0	0
11	0.0000	0.0030	-4.4E-08	0	3.6835E-07	0	4.08E-08	0.002844	0	0
12	0.0000	0.0025	0	0	7.5424E-07	0	4.08E-08	0.002844	0	0
13	0.0000	0.0020	8.27E-08	0	9.5595E-07	0	4.08E-08	0.002844	0	0
14	0.0000	0.0017	1.69E-07	0	7.7178E-07	0	4.08E-08	0.002844	0	0
15	0.0000	0.0014	2.14E-07	0	0	1.25E-05	7.22E-08	0.005257	1.14E-06	0
16	0.0000	0.0012	1.72E-07	0	0	1.25E-05	7.22E-08	0.005257	1.14E-06	0
17	0.0000	0.0012	0	0	0	1.25E-05	7.22E-08	0.005257	1.14E-06	0
18	0.0000	0.0013	-3.1E-07	0	0	1.25E-05	7.22E-08	0.005257	1.14E-06	0
19	0.0000	0.0014	-6.4E-07	0	0	1.25E-05	7.22E-08	0.005257	1.14E-06	0
20	0.0000	0.0016	-8E-07	0	0	0.000145	1.11E-07	0.011035	3.12E-06	0
21	0.0000	0.0018	-6.4E-07	0	5.2446E-06	0.000145	1.11E-07	0.011035	3.12E-06	0
22	0.0000	0.0019	0	0	1.0647E-05	0.000145	1.11E-07	0.011035	3.12E-06	0
23	0.0000	0.0019	1.17E-06	0	1.3427E-05	0.000145	1.11E-07	0.011035	3.12E-06	0
24	0.0000	0.0019	2.37E-06	0	1.0805E-05	0.000145	1.11E-07	0.011035	3.12E-06	0
25	0.0000	0.0017	2.99E-06	0	0	0.0007892	2.78E-07	0.025357	1.01E-05	0
26	0.0000	0.0016	2.41E-06	0	0	0.0007892	2.78E-07	0.025357	1.01E-05	0
27	0.0000	0.0014	0	0	0	0.0007892	2.78E-07	0.025357	1.01E-05	0
28	0.0000	0.0012	-4.4E-06	0	0	0.0007892	2.78E-07	0.025357	1.01E-05	0
29	0.0000	0.0011	-8.9E-06	0	0	0.0007892	2.78E-07	0.025357	1.01E-05	0
30	0.0000	0.0010	-1.1E-05	0	0	0.0027449	4.64E-07	0.053076	2.23E-05	0
31	0.0000	0.0009	-9E-06	0	7.3056E-05	0.0027449	4.64E-07	0.053076	2.23E-05	0
32	0.0000	0.0009	0	0	0.0001483	0.0027449	4.64E-07	0.053076	2.23E-05	0
33	0.0001	0.0010	1.79E-05	0	0.00018702	0.0027449	4.64E-07	0.053076	2.23E-05	0
34	0.0001	0.0011	4.58E-05	0	0.0001505	0.0027449	4.64E-07	0.053076	2.23E-05	0
35	0.0002	0.0013	8.49E-05	0	0	0.007137	9.02E-07	0.09617	4.76E-05	0
36	0.0001	0.0014	0.000136	0	0	0.007137	9.02E-07	0.09617	4.76E-05	0
37	0.0000	0.0014	0.0002	0	0	0.007137	9.02E-07	0.09617	4.76E-05	0
38	0.0000	0.0014	0.000278	0	0.00027401	0.007137	9.02E-07	0.09617	4.76E-05	0
39	0.0000	0.0014	0.000368	0	0.00174234	0.007137	9.02E-07	0.09617	4.76E-05	0
40	0.0000	0.0018	0.000469	0	0.0045	0.015248	1.58E-06	0.151663	0.0001	0
41	0.0004	0.0026	0.00058	0	0.00880314	0.015248	1.58E-06	0.151663	0.0001	0
42	0.0017	0.0041	0.0007	0	0.01449041	0.015248	1.58E-06	0.151663	0.0001	0
43	0.0039	0.0064	0.000828	0	0.02129612	0.015248	1.58E-06	0.151663	0.0001	0
44	0.0067	0.0095	0.000967	0	0.02895455	0.015248	1.58E-06	0.151663	0.0001	0
45	0.0099	0.0130	0.001122	0	0.0372	0.0290239	4E-06	0.21323	0.000172	0
46	0.0133	0.0168	0.001298	0	0.0457565	0.0290239	4E-06	0.21323	0.000172	0
47	0.0165	0.0207	0.0015	0	0.05430704	0.0290239	4E-06	0.21323	0.000172	0
48	0.0193	0.0245	0.00173	0	0.06252432	0.0290239	4E-06	0.21323	0.000172	0
49	0.0217	0.0280	0.001982	0	0.07008107	0.0290239	4E-06	0.21323	0.000172	0
50	0.0235	0.0311	0.00225	0	0.07665	0.0492599	4.35E-06	0.274774	0.000366	0
51	0.0247	0.0335	0.002525	0	0.08197725	0.0492599	4.35E-06	0.274774	0.000366	0
52	0.0252	0.0353	0.0028	0	0.08610265	0.0492599	4.35E-06	0.274774	0.000366	0
53	0.0249	0.0362	0.003068	0	0.08913941	0.0492599	4.35E-06	0.274774	0.000366	0
54	0.0242	0.0365	0.003326	0	0.09120078	0.0492599	4.35E-06	0.274774	0.000366	0
55	0.0230	0.0362	0.003569	0	0.0924	0.0736713	9.78E-06	0.33139	0.000713	0
56	0.0218	0.0357	0.003795	0	0.09284888	0.0736713	9.78E-06	0.33139	0.000713	0
57	0.0206	0.0351	0.004	0	0.09265358	0.0736713	9.78E-06	0.33139	0.000713	0
58	0.0197	0.0346	0.004184	0	0.09191884	0.0736713	9.78E-06	0.33139	0.000713	0
59	0.0191	0.0344	0.004361	0	0.0907494	0.0736713	9.78E-06	0.33139	0.000713	0

	K	L	M	N	O	P	Q	R	S	T
60	0.0187	0.0344	0.004545	0	0.08925	0.0959419	1.22E-05	0.379958	0.001429	0
61	0.0184	0.0348	0.004753	0	0.08752243	0.0959419	1.22E-05	0.379958	0.001429	0
62	0.0183	0.0357	0.005	0	0.08565664	0.0959419	1.22E-05	0.379958	0.001429	0
63	0.0184	0.0371	0.005299	0	0.08373964	0.0959419	1.22E-05	0.379958	0.001429	0
64	0.0185	0.0388	0.005646	0	0.08185842	0.0959419	1.22E-05	0.379958	0.001429	0
65	0.0186	0.0407	0.006033	0	0.0801	0.116313	2.18E-05	0.418721	0.002358	0
66	0.0189	0.0424	0.006454	0	0.07852981	0.116313	2.18E-05	0.418721	0.002358	0
67	0.0192	0.0439	0.0069	0	0.07712707	0.116313	2.18E-05	0.418721	0.002358	0
68	0.0195	0.0449	0.007364	0	0.07584942	0.116313	2.18E-05	0.418721	0.002358	0
69	0.0199	0.0453	0.00783	0	0.07465451	0.116313	2.18E-05	0.418721	0.002358	0
70	0.0203	0.0452	0.008286	0	0.0735	0.1386748	2.83E-05	0.446749	0.00359	0
71	0.0209	0.0444	0.008714	0	0.07235113	0.1386748	2.83E-05	0.446749	0.00359	0
72	0.0215	0.0431	0.0091	0	0.07120349	0.1386748	2.83E-05	0.446749	0.00359	0
73	0.0223	0.0411	0.009431	0	0.0700603	0.1386748	2.83E-05	0.446749	0.00359	0
74	0.0232	0.0387	0.009701	0	0.06892474	0.1386748	2.83E-05	0.446749	0.00359	0
75	0.0242	0.0358	0.009905	0	0.0678	0.1569095	3.26E-05	0.463411	0.004983	0
76	0.0254	0.0327	0.010039	0	0.06669008	0.1569095	3.26E-05	0.463411	0.004983	0
77	0.0266	0.0295	0.0101	0	0.06560216	0.1569095	3.26E-05	0.463411	0.004983	0
78	0.0281	0.0263	0.010085	0	0.06454419	0.1569095	3.26E-05	0.463411	0.004983	0
79	0.0296	0.0234	0.010005	0	0.06352415	0.1569095	3.26E-05	0.463411	0.004983	0
80	0.0313	0.0209	0.009873	0	0.06255	0.1697729	4.26E-05	0.469189	0.006018	0
81	0.0332	0.0191	0.0097	0	0.06163094	0.1697729	4.26E-05	0.469189	0.006018	0
82	0.0353	0.0182	0.0095	0	0.06078107	0.1697729	4.26E-05	0.469189	0.006018	0
83	0.0375	0.0184	0.009286	0	0.06001574	0.1697729	4.26E-05	0.469189	0.006018	0
84	0.0401	0.0199	0.009073	0	0.05935027	0.1697729	4.26E-05	0.469189	0.006018	0
85	0.0434	0.0225	0.008877	0	0.0588	0.1818543	4.42E-05	0.463825	0.006417	0
86	0.0475	0.0264	0.008714	0	0.05837336	0.1818543	4.42E-05	0.463825	0.006417	0
87	0.0527	0.0315	0.0086	0	0.05805114	0.1818543	4.42E-05	0.463825	0.006417	0
88	0.0591	0.0379	0.008546	0	0.05780725	0.1818543	4.42E-05	0.463825	0.006417	0
89	0.0665	0.0452	0.008549	0	0.05761557	0.1818543	4.42E-05	0.463825	0.006417	0
90	0.0747	0.0534	0.0086	0	0.05745	0.187765	4.7E-05	0.455913	0.006157	0
91	0.0747	0.0534	0.008692	0	0.05745	0.187765	4.7E-05	0.455913	0.006157	0
92	0.0747	0.0534	0.008816	0	0.05745	0.187765	4.7E-05	0.455913	0.006157	0
93	0.0747	0.0534	0.008964	0	0.05745	0.187765	4.7E-05	0.455913	0.006157	0
94	0.0747	0.0534	0.009128	0	0.05745	0.187765	4.7E-05	0.455913	0.006157	0
95	0.0747	0.0534	0.0093	0	0.05745	0.187765	4.7E-05	0.455913	0.006157	0
96	0.0747	0.0534	0.0093	0	0.05745	0.187765	4.7E-05	0.455913	0.006157	0
97	0.0747	0.0534	0.0093	0	0.05745	0.187765	4.7E-05	0.455913	0.006157	0
98	0.0747	0.0534	0.0093	0	0.05745	0.187765	4.7E-05	0.455913	0.006157	0
99	0.0747	0.0534	0.0093	0	0.05745	0.187765	4.7E-05	0.455913	0.006157	0
100	0.0747	0.0534	0.0093	0	0.05745	0.187765	4.7E-05	0.455913	0.006157	0
101	0.0747	0.0534	0.0093	0	0.05745	0.187765	4.7E-05	0.455913	0.006157	0
102	0.0747	0.0534	0.0093	0	0.05745	0.187765	4.7E-05	0.455913	0.006157	0
103	0.0747	0.0534	0.0093	0	0.05745	0.187765	4.7E-05	0.455913	0.006157	0
104	0.0747	0.0534	0.0093	0	0.05745	0.187765	4.7E-05	0.455913	0.006157	0
105	0.0747	0.0534	0.0093	0	0.05745	0.187765	4.7E-05	0.455913	0.006157	0
106						From Indigenous BOD (remote)				
107	**Case fatality**					**Prevalence (initial)**				
108	I20-I25: Ischaemic heart diseases	I60-I69: Cerebrovascular diseases	E11,E14: Diabetes	C50: Breast	C18-20: Colorectum	I20-I25: Ischaemic heart diseases	I60-I69: Cerebrovascular diseases	E11,E14: Diabetes	C50: Breast	C18-20: Colorectum
109	0.0000	0.0000	0.0069	0	0	0	8.52E-09	0.000122	0	0
110	0.0000	0.0000	0.006483	0	0	0	4.4E-08	0.000793	0	0
111	0.0000	0.0000	0.006	5.2E-05	1.1072E-07	0	4.4E-08	0.000793	0	0
112	0.0000	0.0000	0.005404	8.33E-05	1.7716E-07	0	4.4E-08	0.000793	0	0
113	0.0000	0.0000	0.004716	7.29E-05	1.5501E-07	0	4.4E-08	0.000793	0	0
114	0.0000	0.0000	0.003977	0	0	0	6.07E-08	0.00235	1.34E-06	0
115	0.0000	0.0001	0.003225	0	0	0	6.07E-08	0.00235	1.34E-06	0
116	0.0000	0.0001	0.0025	0	0	0	6.07E-08	0.00235	1.34E-06	0
117	0.0000	0.0002	0.001837	9.05E-05	0	0	6.07E-08	0.00235	1.34E-06	0

	K	L	M	N	O	P	Q	R	S	T
118	0.0000	0.0002	0.001254	0.000782	0	0	6.07E-08	0.00235	1.34E-06	0
119	0.0000	0.0003	0.000761	0.0021	0	2.5E-06	8.75E-08	0.004184	9.99E-07	0
120	0.0000	0.0004	0.000373	0.00416	1.1338E-06	2.5E-06	8.75E-08	0.004184	9.99E-07	0
121	0.0000	0.0005	0.0001	0.006832	2.303E-06	2.5E-06	8.75E-08	0.004184	9.99E-07	0
122	0.0000	0.0006	-5.1E-05	0.009924	2.9054E-06	2.5E-06	8.75E-08	0.004184	9.99E-07	0
123	0.0000	0.0006	-0.00011	0.013244	2.3385E-06	2.5E-06	8.75E-08	0.004184	9.99E-07	0
124	0.0000	0.0007	-9.5E-05	0.0166	0	7E-05	1.3E-07	0.008475	9.36E-06	0
125	0.0000	0.0007	-4.9E-05	0.019822	0	7E-05	1.3E-07	0.008475	9.36E-06	0
126	0.0000	0.0007	0	0.022825	0	7E-05	1.3E-07	0.008475	9.36E-06	0
127	0.0000	0.0007	2.7E-05	0.025547	0	7E-05	1.3E-07	0.008475	9.36E-06	0
128	0.0000	0.0006	3.32E-05	0.027927	0	7E-05	1.3E-07	0.008475	9.36E-06	0
129	0.0000	0.0006	2.58E-05	0.0299	0	0.000578	6.94E-07	0.022906	1.64E-05	7.46E-06
130	0.0000	0.0006	1.23E-05	0.031496	1.5816E-05	0.000578	6.94E-07	0.022906	1.64E-05	7.46E-06
131	0.0000	0.0006	0	0.033104	3.2108E-05	0.000578	6.94E-07	0.022906	1.64E-05	7.46E-06
132	0.0000	0.0006	-4.6E-06	0.035203	4.0491E-05	0.000578	6.94E-07	0.022906	1.64E-05	7.46E-06
133	0.0000	0.0008	1.28E-06	0.038276	3.2583E-05	0.000578	6.94E-07	0.022906	1.64E-05	7.46E-06
134	0.0000	0.0010	1.94E-05	0.0428	0	0.0024139	5.83E-07	0.054635	2.32E-05	4.12E-05
135	0.0000	0.0014	5.17E-05	0.048989	0	0.0024139	5.83E-07	0.054635	2.32E-05	4.12E-05
136	0.0000	0.0020	0.0001	0.055982	0	0.0024139	5.83E-07	0.054635	2.32E-05	4.12E-05
137	0.0000	0.0028	0.000165	0.06265	0	0.0024139	5.83E-07	0.054635	2.32E-05	4.12E-05
138	0.0000	0.0037	0.000243	0.067866	0	0.0024139	5.83E-07	0.054635	2.32E-05	4.12E-05
139	0.0000	0.0047	0.000328	0.0705	0	0.007066	9.09E-07	0.107171	4.82E-05	0.000154
140	0.0000	0.0056	0.000415	0.069762	0.0003115	0.007066	9.09E-07	0.107171	4.82E-05	0.000154
141	0.0000	0.0063	0.0005	0.066208	0.00117681	0.007066	9.09E-07	0.107171	4.82E-05	0.000154
142	0.0000	0.0067	0.000579	0.060733	0.00302637	0.007066	9.09E-07	0.107171	4.82E-05	0.000154
143	0.0000	0.0070	0.000653	0.054232	0.00629062	0.007066	9.09E-07	0.107171	4.82E-05	0.000154
144	0.0000	0.0070	0.000729	0.0476	0.0114	0.0171846	1.81E-06	0.176967	0.000104	0.00043
145	0.0000	0.0068	0.00081	0.041596	0.01860344	0.0171846	1.81E-06	0.176967	0.000104	0.00043
146	0.0000	0.0066	0.0009	0.036445	0.02742381	0.0171846	1.81E-06	0.176967	0.000104	0.00043
147	0.0000	0.0063	0.001003	0.032235	0.03720245	0.0171846	1.81E-06	0.176967	0.000104	0.00043
148	0.0000	0.0060	0.001116	0.029057	0.04728073	0.0171846	1.81E-06	0.176967	0.000104	0.00043
149	0.0000	0.0056	0.001239	0.027	0.057	0.0358209	2.78E-06	0.253971	0.000198	0.00095
150	0.0000	0.0053	0.001368	0.026102	0.06578034	0.0358209	2.78E-06	0.253971	0.000198	0.00095
151	0.0000	0.0050	0.0015	0.026194	0.07335677	0.0358209	2.78E-06	0.253971	0.000198	0.00095
152	0.0000	0.0048	0.001635	0.027054	0.07954303	0.0358209	2.78E-06	0.253971	0.000198	0.00095
153	0.0000	0.0046	0.001777	0.028463	0.08415286	0.0358209	2.78E-06	0.253971	0.000198	0.00095
154	0.0000	0.0045	0.001931	0.0302	0.087	0.061541	4.05E-06	0.329188	0.000402	0.001566
155	0.0000	0.0046	0.002104	0.032084	0.08800239	0.061541	4.05E-06	0.329188	0.000402	0.001566
156	0.0000	0.0048	0.0023	0.034101	0.0874947	0.061541	4.05E-06	0.329188	0.000402	0.001566
157	0.0001	0.0052	0.002525	0.036276	0.08591583	0.061541	4.05E-06	0.329188	0.000402	0.001566
158	0.0002	0.0058	0.002778	0.038633	0.08370463	0.061541	4.05E-06	0.329188	0.000402	0.001566
159	0.0004	0.0065	0.003059	0.0412	0.0813	0.0907353	1.04E-05	0.396798	0.000659	0.00228
160	0.0006	0.0075	0.003367	0.044004	0.07906771	0.0907353	1.04E-05	0.396798	0.000659	0.00228
161	0.0009	0.0086	0.0037	0.047084	0.07708122	0.0907353	1.04E-05	0.396798	0.000659	0.00228
162	0.0012	0.0099	0.004059	0.050482	0.07534086	0.0907353	1.04E-05	0.396798	0.000659	0.00228
163	0.0016	0.0111	0.004451	0.05424	0.07384701	0.0907353	1.04E-05	0.396798	0.000659	0.00228
164	0.0020	0.0121	0.004883	0.0584	0.0726	0.1218246	1.44E-05	0.453713	0.001078	0.002968
165	0.0023	0.0127	0.005363	0.063	0.07159036	0.1218246	1.44E-05	0.453713	0.001078	0.002968
166	0.0026	0.0126	0.0059	0.068062	0.07076923	0.1218246	1.44E-05	0.453713	0.001078	0.002968
167	0.0028	0.0118	0.006495	0.073604	0.07007792	0.1218246	1.44E-05	0.453713	0.001078	0.002968
168	0.0030	0.0105	0.007129	0.079644	0.06945774	0.1218246	1.44E-05	0.453713	0.001078	0.002968
169	0.0032	0.0089	0.007774	0.0862	0.06885	0.1468557	2E-05	0.499478	0.001811	0.003505
170	0.0036	0.0074	0.008407	0.093288	0.06820526	0.1468557	2E-05	0.499478	0.001811	0.003505
171	0.0043	0.0063	0.009	0.100911	0.06751107	0.1468557	2E-05	0.499478	0.001811	0.003505
172	0.0053	0.0058	0.009532	0.10907	0.06676426	0.1468557	2E-05	0.499478	0.001811	0.003505
173	0.0065	0.0058	0.009993	0.117766	0.06596163	0.1468557	2E-05	0.499478	0.001811	0.003505
174	0.0079	0.0064	0.010378	0.127	0.0651	0.1675932	2.9E-05	0.534973	0.002715	0.003653
175	0.0094	0.0074	0.010682	0.136756	0.06418382	0.1675932	2.9E-05	0.534973	0.002715	0.003653
176	0.0110	0.0087	0.0109	0.146952	0.06324808	0.1675932	2.9E-05	0.534973	0.002715	0.003653
177	0.0126	0.0103	0.01103	0.15749	0.06233544	0.1675932	2.9E-05	0.534973	0.002715	0.003653
178	0.0142	0.0122	0.011087	0.168272	0.06148853	0.1675932	2.9E-05	0.534973	0.002715	0.003653
179	0.0158	0.0144	0.011088	0.1792	0.06075	0.1874922	3.66E-05	0.560569	0.003661	0.003567
180	0.0174	0.0169	0.011053	0.190329	0.06015628	0.1874922	3.66E-05	0.560569	0.003661	0.003567
181	0.0191	0.0198	0.011	0.202329	0.05971899	0.1874922	3.66E-05	0.560569	0.003661	0.003567

	K	L	M	N	O	P	Q	R	S	T
182	0.0209	0.0231	0.010944	0.216025	0.05944357	0.1874922	3.66E-05	0.560569	0.003661	0.003567
183	0.0227	0.0266	0.010893	0.23224	0.05933543	0.1874922	3.66E-05	0.560569	0.003661	0.003567
184	0.0246	0.0305	0.010849	0.2518	0.0594	0.2024279	4.41E-05	0.57583	0.004712	0.003314
185	0.0266	0.0345	0.010817	0.275253	0.05963707	0.2024279	4.41E-05	0.57583	0.004712	0.003314
186	0.0287	0.0386	0.0108	0.302046	0.06002394	0.2024279	4.41E-05	0.57583	0.004712	0.003314
187	0.0311	0.0428	0.0108	0.331353	0.06053227	0.2024279	4.41E-05	0.57583	0.004712	0.003314
188	0.0339	0.0470	0.010811	0.362347	0.06113374	0.2024279	4.41E-05	0.57583	0.004712	0.003314
189	0.0374	0.0512	0.01082	0.3942	0.0618	0.2118478	5.18E-05	0.579671	0.005183	0.003001
190	0.0419	0.0553	0.01082	0.426222	0.06250624	0.2118478	5.18E-05	0.579671	0.005183	0.003001
191	0.0476	0.0593	0.0108	0.458269	0.06324165	0.2118478	5.18E-05	0.579671	0.005183	0.003001
192	0.0547	0.0632	0.010752	0.490335	0.06399895	0.2118478	5.18E-05	0.579671	0.005183	0.003001
193	0.0630	0.0669	0.010679	0.522414	0.06477083	0.2118478	5.18E-05	0.579671	0.005183	0.003001
194	0.0722	0.0706	0.010583	0.5545	0.06555	0.2137978	5.62E-05	0.577581	0.005128	0.002788
195	0.0722	0.0706	0.010469	0.5545	0.06555	0.2137978	5.62E-05	0.577581	0.005128	0.002788
196	0.0722	0.0706	0.01034	0.5545	0.06555	0.2137978	5.62E-05	0.577581	0.005128	0.002788
197	0.0722	0.0706	0.010199	0.5545	0.06555	0.2137978	5.62E-05	0.577581	0.005128	0.002788
198	0.0722	0.0706	0.010051	0.5545	0.06555	0.2137978	5.62E-05	0.577581	0.005128	0.002788
199	0.0722	0.0706	0.0099	0.5545	0.06555	0.2137978	5.62E-05	0.577581	0.005128	0.002788
200	0.0722	0.0706	0.0099	0.5545	0.06555	0.2137978	5.62E-05	0.577581	0.005128	0.002788
201	0.0722	0.0706	0.0099	0.5545	0.06555	0.2137978	5.62E-05	0.577581	0.005128	0.002788
202	0.0722	0.0706	0.0099	0.5545	0.06555	0.2137978	5.62E-05	0.577581	0.005128	0.002788
203	0.0722	0.0706	0.0099	0.5545	0.06555	0.2137978	5.62E-05	0.577581	0.005128	0.002788
204	0.0722	0.0706	0.0099	0.5545	0.06555	0.2137978	5.62E-05	0.577581	0.005128	0.002788
205	0.0722	0.0706	0.0099	0.5545	0.06555	0.2137978	5.62E-05	0.577581	0.005128	0.002788
206	0.0722	0.0706	0.0099	0.5545	0.06555	0.2137978	5.62E-05	0.577581	0.005128	0.002788
207	0.0722	0.0706	0.0099	0.5545	0.06555	0.2137978	5.62E-05	0.577581	0.005128	0.002788
208	0.0722	0.0706	0.0099	0.5545	0.06555	0.2137978	5.62E-05	0.577581	0.005128	0.002788
209	0.0722	0.0706	0.0099	0.5545	0.06555	0.2137978	5.62E-05	0.577581	0.005128	0.002788

	U	V	W	X	Y
1					
2					
3	**2010AUD$ per case**				
4	I20-I25: Ischaemic heart	I60-I69: Cerebrovascular	E11,E14: Diabete	C50: Breast	C18-20: Colorectum
5	$2,962	$2,228	$504		$17,490
6	$2,962	$2,228	$504		$17,490
7	$2,962	$2,228	$504		$17,490
8	$2,962	$2,228	$504		$17,490
9	$2,962	$2,228	$504		$17,490
10	$2,962	$2,228	$504		$17,490
11	$2,962	$2,228	$504		$17,490
12	$2,962	$2,228	$504		$17,490
13	$2,962	$2,228	$504		$17,490
14	$2,962	$2,228	$504		$17,490
15	$2,962	$2,228	$504		$17,490
16	$2,962	$2,228	$504		$17,490
17	$2,962	$2,228	$504		$17,490
18	$2,962	$2,228	$504		$17,490
19	$2,962	$2,228	$504		$17,490
20	$2,962	$2,228	$504		$17,490
21	$2,962	$2,228	$504		$17,490
22	$2,962	$2,228	$504		$17,490
23	$2,962	$2,228	$504		$17,490
24	$2,962	$2,228	$504		$17,490
25	$2,962	$2,228	$504		$17,490
26	$2,962	$2,228	$504		$17,490
27	$2,962	$2,228	$504		$17,490
28	$2,962	$2,228	$504		$17,490
29	$2,962	$2,228	$504		$17,490
30	$2,962	$2,228	$504		$17,490
31	$2,962	$2,228	$504		$17,490
32	$2,962	$2,228	$504		$17,490
33	$2,962	$2,228	$504		$17,490
34	$2,962	$2,228	$504		$17,490
35	$2,962	$2,228	$504		$17,490
36	$2,962	$2,228	$504		$17,490
37	$2,962	$2,228	$504		$17,490
38	$2,962	$2,228	$504		$17,490
39	$2,962	$2,228	$504		$17,490
40	$2,962	$2,228	$504		$17,490
41	$2,962	$2,228	$504		$17,490
42	$2,962	$2,228	$504		$17,490
43	$2,962	$2,228	$504		$17,490
44	$2,962	$2,228	$504		$17,490
45	$2,962	$2,228	$504		$17,490
46	$2,962	$2,228	$504		$17,490
47	$2,962	$2,228	$504		$17,490
48	$2,962	$2,228	$504		$17,490
49	$2,962	$2,228	$504		$17,490
50	$2,962	$2,228	$504		$17,490
51	$2,962	$2,228	$504		$17,490
52	$2,962	$2,228	$504		$17,490
53	$2,962	$2,228	$504		$17,490
54	$2,962	$2,228	$504		$17,490
55	$2,962	$2,228	$504		$17,490
56	$2,865	$2,499	$520		$17,507
57	$2,767	$2,771	$535		$17,523
58	$2,670	$3,042	$551		$17,540
59	$2,573	$3,313	$567		$17,557

	U	V	W	X	Y
60	$2,475	$3,585	$582		$17,573
61	$2,378	$3,856	$598		$17,590
62	$2,280	$4,128	$613		$17,607
63	$2,183	$4,399	$629		$17,623
64	$2,086	$4,670	$645		$17,640
65	$1,988	$4,942	$660		$17,657
66	$1,956	$5,400	$671		$17,707
67	$1,923	$5,859	$681		$17,758
68	$1,891	$6,318	$691		$17,809
69	$1,859	$6,776	$701		$17,860
70	$1,826	$7,235	$711		$17,910
71	$1,794	$7,694	$722		$17,961
72	$1,761	$8,152	$732		$18,012
73	$1,729	$8,611	$742		$18,063
74	$1,697	$9,070	$752		$18,113
75	$1,664	$9,529	$763		$18,164
76	$1,649	$9,861	$750		$18,151
77	$1,634	$10,194	$738		$18,139
78	$1,619	$10,527	$726		$18,126
79	$1,603	$10,860	$713		$18,113
80	$1,588	$11,192	$701		$18,101
81	$1,573	$11,525	$688		$18,088
82	$1,558	$11,858	$676		$18,075
83	$1,543	$12,191	$664		$18,062
84	$1,527	$12,524	$651		$18,050
85	$1,512	$12,856	$639		$18,037
86	$1,500	$13,201	$634		$18,162
87	$1,489	$13,545	$630		$18,287
88	$1,477	$13,890	$625		$18,412
89	$1,465	$14,234	$621		$18,538
90	$1,453	$14,579	$616		$18,663
91	$1,441	$14,923	$612		$18,788
92	$1,430	$15,268	$607		$18,913
93	$1,418	$15,612	$603		$19,038
94	$1,406	$15,956	$598		$19,163
95	$1,394	$16,301	$594		$19,288
96	$1,394	$16,301	$594		$19,288
97	$1,394	$16,301	$594		$19,288
98	$1,394	$16,301	$594		$19,288
99	$1,394	$16,301	$594		$19,288
100	$1,394	$16,301	$594		$19,288
101	$1,394	$16,301	$594		$19,288
102	$1,394	$16,301	$594		$19,288
103	$1,394	$16,301	$594		$19,288
104	$1,394	$16,301	$594		$19,288
105	$1,394	$16,301	$594		$19,288
106					
107	**2010AUD$ per case**				
108	I20-I25: Ischaemic heart diseases	I60-I69: Cerebrovascular diseases	E11,E14: Diabetes	C50: Breast	C18-20: Colorectum
109	$1,832	$1,161	$506	$12,424	$17,136
110	$1,832	$1,161	$506	$12,424	$17,136
111	$1,832	$1,161	$506	$12,424	$17,136
112	$1,832	$1,161	$506	$12,424	$17,136
113	$1,832	$1,161	$506	$12,424	$17,136
114	$1,832	$1,161	$506	$12,424	$17,136
115	$1,832	$1,161	$506	$12,424	$17,136
116	$1,832	$1,161	$506	$12,424	$17,136
117	$1,832	$1,161	$506	$12,424	$17,136

	U	V	W	X	Y
118	$1,832	$1,161	$506	$12,424	$17,136
119	$1,832	$1,161	$506	$12,424	$17,136
120	$1,832	$1,161	$506	$12,424	$17,136
121	$1,832	$1,161	$506	$12,424	$17,136
122	$1,832	$1,161	$506	$12,424	$17,136
123	$1,832	$1,161	$506	$12,424	$17,136
124	$1,832	$1,161	$506	$12,424	$17,136
125	$1,832	$1,161	$506	$12,424	$17,136
126	$1,832	$1,161	$506	$12,424	$17,136
127	$1,832	$1,161	$506	$12,424	$17,136
128	$1,832	$1,161	$506	$12,424	$17,136
129	$1,832	$1,161	$506	$12,424	$17,136
130	$1,832	$1,161	$506	$12,424	$17,136
131	$1,832	$1,161	$506	$12,424	$17,136
132	$1,832	$1,161	$506	$12,424	$17,136
133	$1,832	$1,161	$506	$12,424	$17,136
134	$1,832	$1,161	$506	$12,424	$17,136
135	$1,832	$1,161	$506	$12,424	$17,136
136	$1,832	$1,161	$506	$12,424	$17,136
137	$1,832	$1,161	$506	$12,424	$17,136
138	$1,832	$1,161	$506	$12,424	$17,136
139	$1,832	$1,161	$506	$12,424	$17,136
140	$1,832	$1,161	$506	$12,424	$17,136
141	$1,832	$1,161	$506	$12,424	$17,136
142	$1,832	$1,161	$506	$12,424	$17,136
143	$1,832	$1,161	$506	$12,424	$17,136
144	$1,832	$1,161	$506	$12,424	$17,136
145	$1,832	$1,161	$506	$12,424	$17,136
146	$1,832	$1,161	$506	$12,424	$17,136
147	$1,832	$1,161	$506	$12,424	$17,136
148	$1,832	$1,161	$506	$12,424	$17,136
149	$1,832	$1,161	$506	$12,424	$17,136
150	$1,832	$1,161	$506	$12,424	$17,136
151	$1,832	$1,161	$506	$12,424	$17,136
152	$1,832	$1,161	$506	$12,424	$17,136
153	$1,832	$1,161	$506	$12,424	$17,136
154	$1,832	$1,161	$506	$12,424	$17,136
155	$1,832	$1,161	$506	$12,424	$17,136
156	$1,832	$1,161	$506	$12,424	$17,136
157	$1,832	$1,161	$506	$12,424	$17,136
158	$1,832	$1,161	$506	$12,424	$17,136
159	$1,832	$1,161	$506	$12,424	$17,136
160	$1,800	$1,254	$531	$12,231	$17,058
161	$1,769	$1,347	$556	$12,038	$16,979
162	$1,738	$1,440	$582	$11,845	$16,900
163	$1,707	$1,533	$607	$11,652	$16,822
164	$1,676	$1,626	$632	$11,459	$16,743
165	$1,645	$1,719	$658	$11,266	$16,664
166	$1,614	$1,812	$683	$11,073	$16,585
167	$1,582	$1,904	$708	$10,880	$16,507
168	$1,551	$1,997	$734	$10,687	$16,428
169	$1,520	$2,090	$759	$10,493	$16,349
170	$1,528	$2,392	$767	$10,605	$16,438
171	$1,535	$2,693	$775	$10,717	$16,527
172	$1,542	$2,995	$783	$10,828	$16,616
173	$1,550	$3,297	$791	$10,940	$16,705
174	$1,557	$3,598	$799	$11,051	$16,794
175	$1,565	$3,900	$807	$11,163	$16,883
176	$1,572	$4,201	$815	$11,274	$16,972
177	$1,580	$4,503	$823	$11,386	$17,061
178	$1,587	$4,805	$831	$11,497	$17,149
179	$1,595	$5,106	$839	$11,609	$17,238
180	$1,592	$5,909	$830	$11,719	$17,250
181	$1,589	$6,712	$820	$11,828	$17,263

	U	V	W	X	Y
182	$1,586	$7,515	$811	$11,938	$17,275
183	$1,582	$8,318	$801	$12,048	$17,287
184	$1,579	$9,121	$792	$12,157	$17,299
185	$1,576	$9,924	$782	$12,267	$17,311
186	$1,573	$10,727	$773	$12,377	$17,323
187	$1,570	$11,530	$763	$12,486	$17,336
188	$1,567	$12,333	$754	$12,596	$17,348
189	$1,564	$13,137	$745	$12,706	$17,360
190	$1,575	$13,791	$713	$12,687	$17,278
191	$1,585	$14,445	$681	$12,669	$17,197
192	$1,596	$15,099	$650	$12,650	$17,116
193	$1,606	$15,753	$618	$12,632	$17,034
194	$1,617	$16,408	$587	$12,613	$16,953
195	$1,628	$17,062	$555	$12,594	$16,871
196	$1,638	$17,716	$523	$12,576	$16,790
197	$1,649	$18,370	$492	$12,557	$16,708
198	$1,660	$19,024	$460	$12,539	$16,627
199	$1,670	$19,679	$429	$12,520	$16,545
200	$1,670	$19,679	$429	$12,520	$16,545
201	$1,670	$19,679	$429	$12,520	$16,545
202	$1,670	$19,679	$429	$12,520	$16,545
203	$1,670	$19,679	$429	$12,520	$16,545
204	$1,670	$19,679	$429	$12,520	$16,545
205	$1,670	$19,679	$429	$12,520	$16,545
206	$1,670	$19,679	$429	$12,520	$16,545
207	$1,670	$19,679	$429	$12,520	$16,545
208	$1,670	$19,679	$429	$12,520	$16,545
209	$1,670	$19,679	$429	$12,520	$16,545
